# Hydrogels from Protein–Polymer Conjugates: A Pathway to Next-Generation Biomaterials

**DOI:** 10.3390/gels11020096

**Published:** 2025-01-29

**Authors:** Oubadah Alayoubi, Yağmur Poyraz, Gana Hassan, Sümeyye Berfin Gül, Nergiz Çalhan, Naz Mina Mert Şahin, Megha Gautam, Aylin Kutlu, Bengü Özuğur Uysal, Ebru Demet Akten, Önder Pekcan

**Affiliations:** 1 Materials Science and Nanotechnology, School of Graduate Studies, Kadir Has University, Cibali, Fatih, Istanbul 34083, Türkiye; 2 Computational Sciences and Engineering, School of Graduate Studies, Kadir Has University, Cibali, Fatih, Istanbul 34083, Türkiye; 3 Department of Basic Sciences, Faculty of Engineering and Architecture, Altınbaş University, Bağcılar, Istanbul 34218, Türkiye; 4 Faculty of Engineering and Natural Sciences, Kadir Has University, Cibali, Fatih, Istanbul 34083, Türkiyepekcan@khas.edu.tr (Ö.P.)

**Keywords:** hybrid hydrogels, protein–polymer conjugates, PEGylation

## Abstract

Hybrid hydrogels from protein–polymer conjugates are biomaterials formed via the chemical bonding of a protein molecule with a polymer molecule. Protein–polymer conjugates offer a variety of biological properties by combining the mechanical strength of polymers and the bioactive functionality of proteins. These properties allow these conjugates to be used as biocompatible components in biomedical applications. Protein–polymer conjugation is a vital bioengineering strategy in many fields, such as drug delivery, tissue engineering, and cancer therapy. Protein–polymer conjugations aim to create materials with new and unique properties by combining the properties of different molecular components. There are various ways of creating protein–polymer conjugates. PEGylation is one of the most common conjugation techniques where a protein is conjugated with Polyethylene Glycol. However, some limitations of PEGylation (like polydispersity and low biodegradability) have prompted researchers to devise novel synthesis techniques like PEGylation, where synthetic polypeptides are used as the polymer component. This review will illustrate the properties of protein–polymer conjugates, their synthesis methods, and their various biomedical applications.

## 1. Introduction

As solitary materials, proteins are a remarkably versatile component in various biomedical applications. Proteins exhibit outstanding biological and economic advantages [1]. In addition to their impressive capability to perform various biological functions, proteins have a high reaction specificity, minimizing their off-target influence. Still, some practical drawbacks limit proteins’ biomedical use. The magnitude and amphiphilicity of proteins may preclude their access to the targeted cell. Also, protease and degrading chemicals may shorten their life span [1,2]. Conjugating proteins and polymers are a highly efficient approach to circumventing complications. The conjugation process transfers the properties of the conjugated polymer to the protein, thus improving its efficiency.

The conjugation of Polyethylene Glycol to protein (a process known as PEGlyation) is an example of a bioconjugation process that can render functional conjugates with the best properties of both its components, prolonging the half-life by inhibiting proteolysis through steric repulsion, as well as decreasing the immunological reaction of the conjugated protein [1,2,3,4]. For example, the PEGylation of the blue-pigment protein Phycocyanin improved the protein’s intensity against thermal and pH changes [5]. Protein–polymer conjugates can form hybrid hydrogels that incorporate the mechanical strength of polymers and the bioactive functionality of proteins. The superior biocompatibility and biodegradability of protein–polymer hydrogels distinguish them as a prime choice in various biomedical applications. For example, gelatin-hyaluronic acid hydrogel forms the matrix for articular cartilage regeneration because its unique structure resembles Extracellular Matrix (ECM) architecture [4,6].

**Figure 1:**
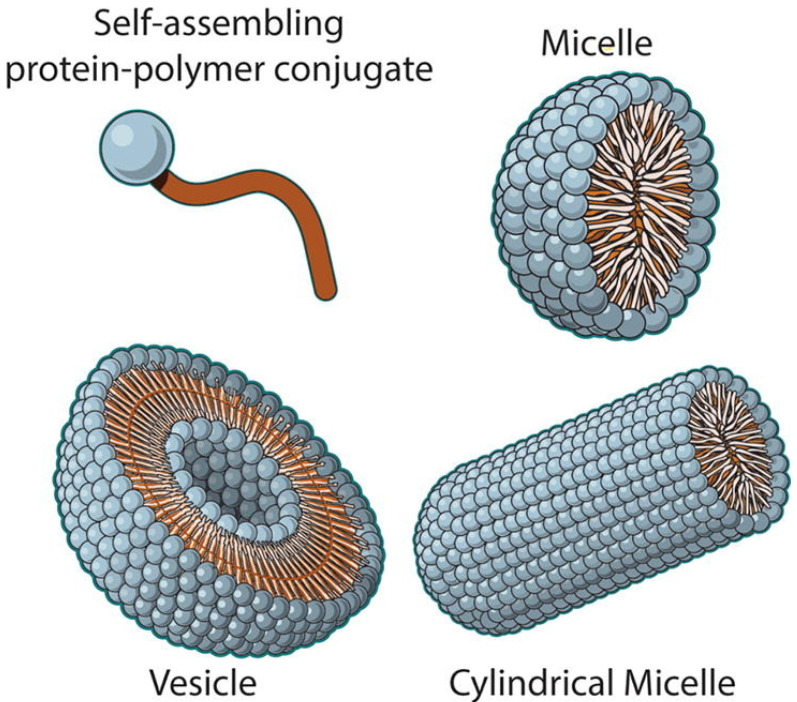
A representation of self-assembly. This schematic illustrates the self-assembly process of amphiphilic block copolymer–protein conjugates into higher-order structures such as micelles, vesicles, and bilayers. The hydrophobic polymer segment associates to form a core, while the hydrophilic protein forms a corona, enabling encapsulation and controlled release of hydrophobic drugs [2].

The conjugation of a hydrophobic polymer with a hydrophilic protein produces an amphiphilic protein–polymer conjugate. Amphiphilic block copolymers can self-assemble into various organized structures. This self-assembled protein can serve as an excellent tunable device of drug delivery that can be designed for temporal and distribution control. Currently, amphiphilic block copolymer protein–polymer conjugates which show great promise are the focus of advanced drug delivery methods. These bioconjugates can be designed to connect hydrophobic polymer to a hydrophilic protein in order to achieve amphiphilicity. These amphiphiles have the ability to self-assemble into a variety of higher-order structures, including micelles, vesicles, and bilayers (Figure 1). Self-assembled protein–polymer conjugates have clear advantages over their monomeric counterparts in drug administration, including distribution management, timing control, and drug encapsulation. Self-assembled protein–polymer conjugates can decrease toxicity, allow sustained release, protect hydrophobic medicines from hydrolysis and other enzymatic or chemical destruction, and increase their bioavailability [2].

Protein–polymer conjugates are versatile carriers for a wide range of drugs, including hydrophobic small molecules, peptides, proteins, and nucleic acids. Their economic advantages stem from their ability to enhance drug efficacy and stability, which reduces the required dosage, minimizes side effects, and lowers overall treatment costs. These conjugates exhibit significantly extended half-lives, ranging from several days to weeks, depending on the specific system and administration conditions. Validating their biomedical use requires comprehensive analyses, including cytotoxicity assays, stability evaluations, pharmacokinetic profiling, biodistribution studies, and immunogenicity assessments, to ensure their safety and efficacy. While strategies like PEGylation effectively mitigate immunological reactions, there is still a possibility of residual immunogenicity or hypersensitivity, necessitating further refinement and rigorous testing to optimize these systems for clinical applications.

Protein–polymer conjugates are integral to hydrogel design, offering a unique combination of mechanical strength and bioactivity. Hydrogels formed by these conjugates mimic the extracellular matrix (ECM) in tissue engineering, promoting cell adhesion, proliferation, and differentiation. For example, gelatin-methacrylate (GelMA)-based hydrogels have been successfully applied in 3D bioprinting for regenerative medicine. In drug delivery, protein–polymer hydrogels provide a controlled release of therapeutic agents, minimizing burst release and achieving sustained delivery over extended periods. Amphiphilic protein–polymer conjugates self-assemble into nanostructures within hydrogels, enabling targeted and environmentally responsive release of hydrophobic drugs. By combining physical gelation with chemical crosslinking, these systems achieve tunable mechanical and degradation properties, making them ideal for applications in wound healing and injectable therapies.

While polymer-based biomedical applications have been extensively reviewed, this manuscript distinguishes itself by integrating the unique role of protein–polymer conjugates in the formation of hybrid hydrogels for next-generation biomaterials. Unlike traditional reviews, this work focuses on correlating advanced polymerization techniques such as Atom Transfer Radical Polymerization (ATRP) and Reversible Addition-Fragmentation Chain Transfer (RAFT) polymerization to specific biomedical functionalities. These methods enable fine-tuning of mechanical properties, degradation rates, and bioactivity. For example, ATRP allows precise control over polymer chain length and end-group functionality, crucial for designing hydrogels with targeted drug-release profiles. RAFT polymerization, with its versatile chain-transfer agents, facilitates the synthesis of biodegradable polymers suitable for tissue scaffolding and regenerative medicine. This approach bridges the gap between synthesis strategies and their application, providing insights into the customization of polymer–protein hydrogels for therapeutic and diagnostic use.

## 2. Synthesis of Protein–Polymer Conjugates

The first protein–polymer conjugate was formed by conjugating monomethoxy-PEG (mPEG) and bovine serum albumin. In animal models, it is less immunogenic than natural protein and prolonged blood residence time [7]. Polyethylene glycol (PEG) is the most widely used polymer in protein–polymer conjugates due to its exceptional solubility, biocompatibility, and ability to extend the half-life of therapeutic proteins. For example, the PEGylation of bovine serum albumin has demonstrated prolonged blood residence time and reduced immunogenicity. Additionally, the PEGylation of the blue-pigment protein Phycocyanin enhanced stability under thermal and pH changes. However, the limitations of PEG, such as its non-biodegradability and polydispersity, have driven the exploration of alternative polymers. In addition, an antitumor chromoprotein neocarzinostatin conjugate (zinostatin stimalamer) is made with a styrene-maleic acid copolymer licensed from the Ministry of Health of Japan [8]. N-(2-hydroxypropyl) methacrylamide (HPMA), polyvinyl prolidon (PVP), poly(2-oxazolines), dextran, polyglutamic acid (PGA), and hydroxyethyl starch (HES) are other polymers whose biological activities have been investigated and they offer unique advantages and challenges. These polymers can be branched, zwitterionic, single or double as a side chain, dendritic, or conjugated as glycopolymer [9]. HPMA-based conjugates provide improved drug delivery capabilities but may require complex synthesis methods. Dextran is highly biocompatible and biodegradable, making it suitable for drug carriers, though it may lack mechanical strength. PGA, biodegradable by lysosomal enzymes, has shown potential in cancer treatments but faces scalability limitations.

A comparison table (Table 1) is summarized as follows:

Table 2 provides a comprehensive overview of various synthesis methods for protein–polymer conjugates, highlighting their techniques, advantages, limitations, and biomedical applications. Controlled Radical Polymerization (CRP) offers precise polymer growth with tunable properties but requires specific initiators, while ATRP and RAFT provide enhanced control with respective challenges in catalyst cost and transfer agent optimization. Grafting strategies, including grafting-to, -from, and -through, enable tailored polymer attachment with unique benefits like site specificity but face hurdles like steric hindrance or initiation complexity. PEGylation methods, such as classical, bridging, and enzymatic PEGylation, improve protein stability and bioavailability, though issues like polydispersity and enzyme availability persist. Site-specific conjugation techniques, including cysteine- and lysine-targeted methods, enhance bioactivity control, paving the way for advanced drug delivery systems and responsive hydrogels.

### 2.1. Controlled Radical Polymerization (CRP)

Before the inception of the conjunction process, researchers applied controlled radical polymerization techniques to define the properties of the protein–polymer conjugate. The CRP techniques help reduce the heterogeneity of protein–polymer conjugates. The following CRP methods (ATRP, RAFT) synthesize well-defined polymers with functionalized end groups, thus preparing the polymer for direct conjugation with protein without the necessity of post-polymerization modifications of end groups [10]. ATRP’s advantage lies in its ability to produce polymers with low polydispersity through a reversible redox reaction. However, it requires expensive catalysts and controlled conditions. RAFT polymerization, while more cost-effective and versatile, involves the use of chain transfer agents that may affect reaction efficiency. Both techniques demonstrate high compatibility with drugs, as evidenced by successful conjugations preserving protein bioactivity and stability.

#### 2.1.1. Atom Transfer Radical Polymerization (ATRP)

**Figure 2:**
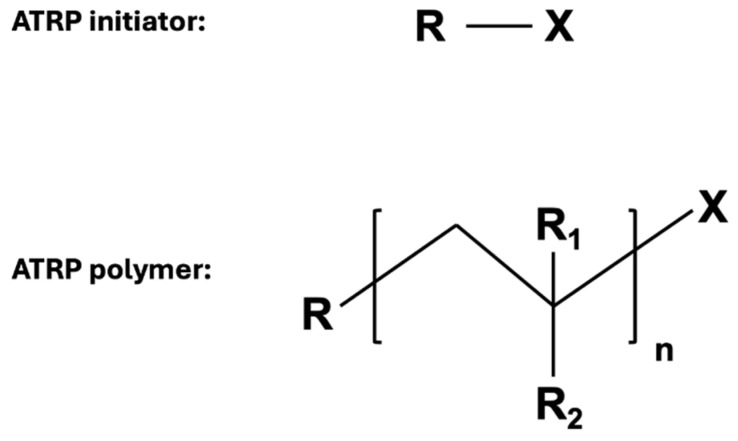
ATRP initiator and polymer. This diagram shows the ATRP process, where a halogenated initiator reacts with a transition metal catalyst to generate a polymerizing radical. The process allows precise control of polymer molecular weight and narrow polydispersity. ATRP-produced polymers are functionalized with specific groups for direct conjugation to proteins, making them suitable for forming bioconjugates with enhanced pharmacokinetic profiles. Permission [11].

This polymerization technique synthesizes a polymer from a halogenated molecule (i.e., initiator). This polymerization initiator can react with a transition metal catalyst. This reversible redox reaction generates a reactive radical central. The polymers formed using this method have a low PDI and carry the initiator at the α-chain and the halogen at the Ω-chain end (Figure 2) [11].

**Figure 3:**
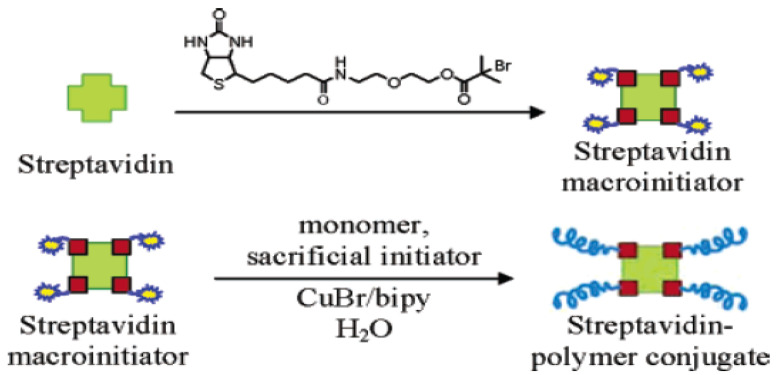
Streptavidin-initiated polymerization. The polymerization of N-isopropylacrylamide (NIPAAm) from streptavidin using ATRP shows site-specific polymer growth. This example illustrates how controlled radical polymerization preserves protein bioactivity while integrating polymer chains for drug delivery applications. The redox-based initiation process is depicted, highlighting the reversible deactivation mechanism [12] American Chemical Society.

Bontempo and his coworkers employed the ATRP technique to conjugate N-isopropyl acrylamide (NIPAAm) polymer with Streptavidin (Sav) protein in one experiment [12]. Their experiment aimed to initiate the polymerization process from a specific domain of the modified sav protein. They reported that ATRP was the optimal CRP method that will help to determine the polymerization initiation location. Their effort remarkably succeeded in initiating the NIPAAm polymer on Sav protein while preserving its bioactivity (Figure 3) [12].

#### 2.1.2. Reversible Addition-Fragmentation Chain Transfer (RAFT)

**Figure 4:**
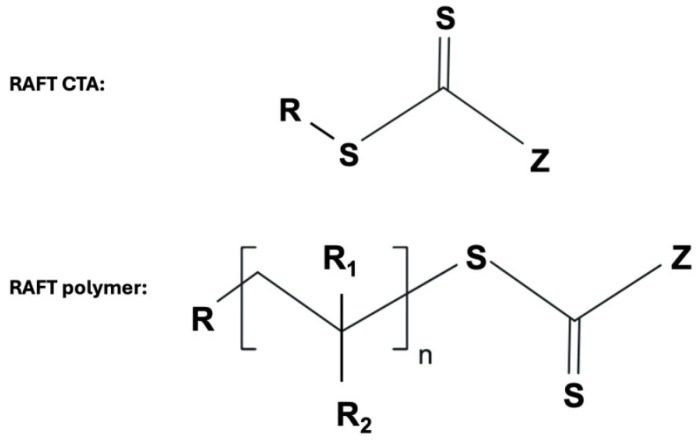
RAFT initiator and polymer. The schematic details RAFT polymerization with a chain transfer agent (CTA), showing how thiocarbonylthio groups mediate controlled polymer growth. The example uses bovine serum albumin (BSA) conjugated with a poly(NIPAAm) chain, demonstrating retained protein functionality and precision in polymer length control. RAFT is critical for synthesizing responsive protein–polymer systems.

This type of polymerization depends on a sacrificial initiator like 2,2′-azobis (2-methylpropionitrile) (AIBN) and a reversible chain transfer agent (CTA) containing thiocarbonylthio group as a mediator. The thiocarbonylthio group attaches to the Ω-chain end of the produced polymer while its α-chain end holds the polymer growth site of the CTA (Figure 4) [11].

**Figure 5:**
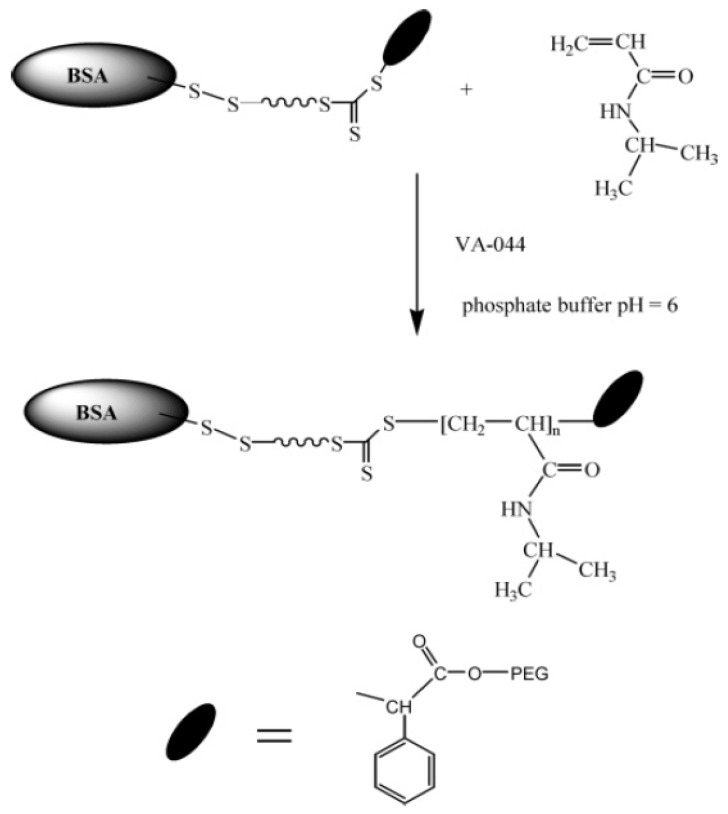
BSA-poly (NIPAAm) conjugation. This figure illustrates the synthesis of a BSA-poly (NIPAAm) conjugate via aqueous RAFT polymerization. The BSA acts as a macroRAFT agent, enabling direct polymer growth from its surface. Applications include responsive hydrogels and smart drug delivery systems that exploit the thermal behavior of NIPAAm-based polymers [13].

Boyer et al. applied the aqueous RAFT technique to polymerize NIPAAm (N-isopropyl acrylamide) and HEA (hydroxyethyl acrylate) polymers. They conducted their experiment using room temperature azo-initiator and BSA-mcroRAFT to synthesize BSA-poly (NIPAAm) and BSA-poly (HEA). BSA-macroRAFT technique preserved the BSA protein’s structure and conformational activity following the conjugation (Figure 5) [13].

### 2.2. Conjugation Methods for the Synthesis of Protein–Polymer Conjugates

In the synthesis of protein–polymer conjugates, the following three basic methods of conjugation were used [14]:

#### 2.2.1. Grafting Polymers to Proteins

**Figure 6:**
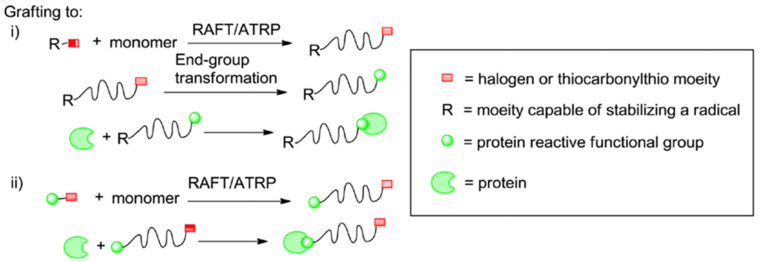
Grafting polymers to proteins technique. The figure shows the grafting-to technique where pre-formed polymers with reactive end groups are conjugated to proteins. This method uses post-polymerization modifications to attach functional polymers, enhancing stability and half-life for therapeutic proteins. Limitations include steric hindrance at high protein concentrations [11].

It is the most widely used conjugation method. This technique obtains the conjugate by connecting the polymer with a previously synthesized functional end group and the complementary functional group on the biomacromolecule under appropriate reaction conditions. High yields are expected from this reaction [11]. It requires the post-polymerization modification of the polymers’ end group (Figure 6):

➢ For polymers synthesized with ATRP, a nucleophile can replace the halogen attached at the Ω-chain end, thus forming a semi-telechelic polymer apt for conjugation.
➢ Polymers synthesized with RAFT can undergo two different paths of modification targeting the thiocarbonylthio groups as follows:

- Radical coupling with functionalized azoinitiators.
- The use of reducing agents (e.g., Sodium Borohydride) of a nucleophile (e.g., Butylamine) to reduce thiocarbonylthio groups to free thiols [11].

#### 2.2.2. Grafting Polymers from Proteins

**Figure 7:**
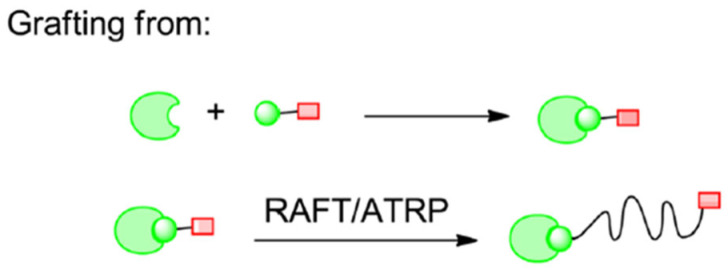
Grafting polymers from proteins. In this method, polymerization initiates directly from functional groups on proteins, producing site-specific protein–polymer conjugates. The diagram shows polymer chains growing from initiator-labeled protein regions, yielding well-defined conjugates suitable for amphiphilic nanostructures [11].

In this conjugation approach, polymerization is performed over certain regions on the biomacromolecule, such as the initiator or chain transfer agent that forms radicals. The protein acts as a macroinitiator. For this method to work, the starting agent of the polymerization must be placed in the biological molecule. To apply this process, chemical conjugation with controlled radical polymerization is essential. The resultant products can easily be purified and characterized and exhibit pharmacokinetic properties superior to unconjugated proteins (Figure 7) [11]. This approach allows the researchers to synthesize conjugates with hydrophobic polymers and obviates the need for separating unreacted polymers and proteins from conjugates [11].

#### 2.2.3. Grafting Through Method

**Figure 8:**
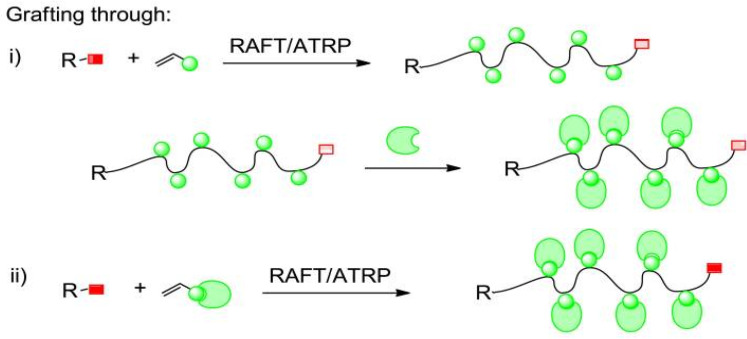
Biomolecules act as monomers during polymerization in the grafting-through technique. The figure illustrates two approaches: (i) Functionalized biomolecules polymerize with monomers via RAFT/ATRP to form linear polymer chains, which further assemble into complex conjugates through biomolecule-specific interactions; and (ii) biomolecules participate directly in polymerization, leading to integrated polymer networks. **Red** represents functional groups or reactive sites on the biomolecule or polymer, essential for initiating polymerization or conjugation. **Green** represents biomolecules, such as proteins or DNA, highlighting their role as biological monomers in the process. This technique is particularly useful for constructing DNA–protein and protein–polymer networks [11].

The biomacromolecule, which acts as a monomer thanks to the polymerization-prone groups on it, can polymerize with different types of monomers to form a conjugate. DNA-polymer and protein–polymer conjugates can be synthesized using these methods (Figure 8) [11].

Various functionalization methods can be applied to the synthesis of conjugates. Depending on functionalization, the conjugation procedures vary. Some of these procedures bind with amine and thiol groups or bind with biotin and streptavidin. Amines are the most targeted functional groups in protein–polymer conjugates. For this type of conjugation, amine groups react with activated esters. The two macromolecules are bound together by the amide bonds formed because of this reaction.

Another frequently preferred method is bonding with thiols. This method aims to establish a bond with the thiol groups in the cysteine amino acid. A disulfide bond forms this bond because of oxidation between two thiols or by exchange with an activated disulfide. Thiols can also form thioether bonds by reacting with alkenes and alkynes. Conjugates can be synthesized using non-covalent interactions such as biotin and avidin binding. The strong and stable affinity of biotin to avidin and streptavidin proteins enables this specific interaction to be used for conjugation [14].

In the direct grafting method, where a biomolecule is polymerized, ensuring the efficiency and efficacy of polymerization is critical. The efficiency and efficacy of polymerization in the direct grafting method depend on factors such as the reaction conditions, the compatibility of the biomolecule with the polymerization initiators, and the control over the polymer chain growth. Techniques like controlled radical polymerization (e.g., ATRP or RAFT) enhance efficiency by minimizing side reactions and ensuring uniform polymer growth. High yields are often achieved when reaction conditions, such as temperature, solvent, and initiator concentration, are optimized. Efficacy is demonstrated by the successful retention of the biomolecule’s structural integrity and functional activity after polymerization. To validate these outcomes, advanced characterization techniques are essential. Nuclear magnetic resonance (NMR) spectroscopy confirms the chemical structure and functionalization of the conjugate. Gel permeation chromatography (GPC) provides insights into molecular weight distribution, indicating uniform polymerization. Electrophoresis verifies the integrity of the biomolecule and its conjugation with the polymer, while mass spectrometry confirms the molecular composition. Together, these techniques ensure that the direct grafting process achieves both high efficiency and efficacy while preserving the intended functionality of the conjugates [15].

### 2.3. Albumin–Polymer Conjugates

The binding of polymers to the albumin protein by physical or chemical means increases the stability of the albumin. Still, there may be changes in the solubility and biocompatibility of the protein depending on the properties of the conjugated polymer. For example, a study has proven that because of the conjugation of albumin with a highly hydrophilic polymer such as polyethyleneglycol (PEG), the conjugate circulates in the blood longer than natural albumin, and its immunogenicity is lower. This protein can be easily conjugated with polymers thanks to functional groups such as thiol, amino, and carboxyl on the albumin surface. Generally, the lysine and cysteine amino acids of albumin are chosen as targets for conjugation with polymers. The amino groups of the lysine amino acid on the albumin surface can be conjugated by reacting with activated esters such as N-hydroxysuccinimide (NHS), with structures such as carboxylic acids and aldehydes. Cysteine-34, which has this free thiol group, can be used effectively for conjugate synthesis. Maleimide and active disulfide groups are functional groups used to interact with the free thiol group of albumins [16].

### 2.4. Polyethylene Glycol (PEG)

**Figure 9:**
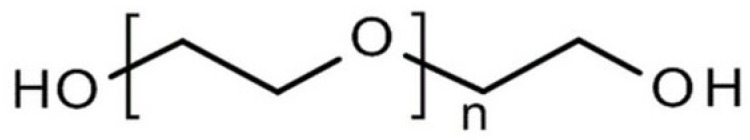
Chemical structure of polyethylene glycol. The structural formula of linear and branched PEG shows repeating ethylene glycol units. PEG is widely used in protein conjugation for its biocompatibility, solubility enhancement, and ability to increase circulation time. Variations in molecular weight and structure influence PEG’s pharmacokinetics and degradation properties.

PEG, a natural polyether with different molecular weights synthesized as linear or branched (Figure 9), has a high solubility in water and many organic solvents. It is widely used in the fields of pharmaceutical biotechnology and bioengineering. The main reason for this is the ability of PEG to remove other polymers in aqueous media. Thus, it does not show immunogenicity and antigenicity by removing proteins. It is non-toxic as it does not damage active proteins and cell membranes [17].

PEGylation methods include classical PEGylation (free cysteines and N-terminal), bridging PEGylation (disulfide bonds), enzymatic PEGylation (glutamines and C-terminal), and glycoPEGylation (O- and N-glycosylation sites or glycans in glycoproteins). With these methods, a single isomer can be formed by increasing site-specific PEGylation and homogeneity [18].

PEG is excreted as a whole from the body via the kidneys (PEGs < 30 kDa) or feces (PEGs > 20 kDa) [19]. Pegylation is defined as modifying proteins, peptides, or other molecules by linking one or more PEG chains. After the pegylation process, the biological properties of the molecules, such as enzymatic activity or recognition by the receptor, do not change. The pegylation process is generally formed from amino groups of proteins by acylation and alkylation reactions. This is because amino groups are active as nucleophiles and are located on the surface of proteins. Pegylation can also be performed from thiol, hydroxyl, and amide groups using specific chemical and enzymatic reactions [20]. For it to bind to a molecule (polypeptide, polysaccharide, polynucleotide, and small organic molecules), it is necessary to prepare the PEG derivative by activating it to contain functional groups at one or both terminals of the PEG. The functional group is selected according to the type of reactive group present in the molecule, and which will combine with the PEG. Reactive amino acids for proteins are lysine, cysteine, histidine, arginine, aspartic acid, glutamic acid, serine, threonine, tyrosine, N-terminal amino group, and C-terminal carboxylic acid. In glycoproteins, the adjacent hydroxyl groups are oxidized with periodate to form two reactive formyl (-CHO) groups [21].

PEG derivatives, such as succinimidyl PEG and PEG-triazine, are widely used in polymerization with bioactive compounds due to their high reactivity and ability to form stable conjugates. The polymerization efficiency with bioactive compounds is influenced by factors like the choice of functional groups, reaction conditions, and the steric hindrance around the active site of the compound. Site-specific conjugation strategies, such as targeting amine or thiol groups on proteins, can enhance efficiency by reducing non-specific interactions. However, certain risks are associated with this process. For instance, the formation of inactive by-products or alterations to the bioactive compound’s functional structure during polymerization may reduce its efficacy. Additionally, some PEG derivatives may induce unwanted immunogenicity or hypersensitivity in specific cases. To mitigate these risks, reaction conditions must be carefully optimized, and site-specific, mild conjugation methods should be employed to preserve the bioactivity of the compound while ensuring high polymerization efficiency.

#### 2.4.1. PEG Derivatives

**Figure 10:**
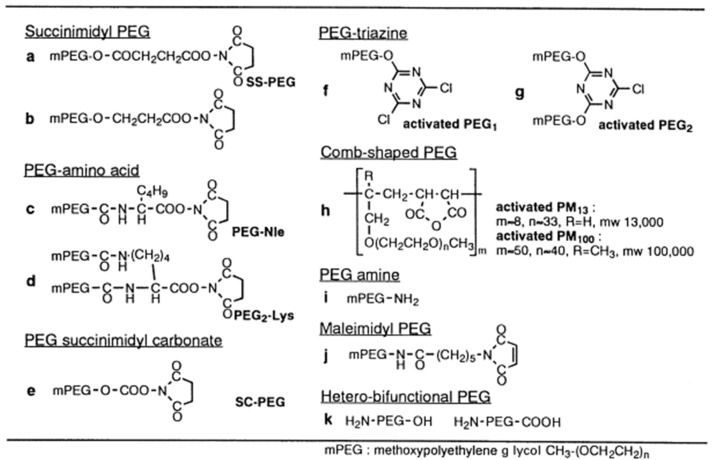
This figure illustrates various PEG derivatives and their functional groups used for modifying proteins and bioactive molecules. (**a**) Succinimidyl PEG reacts with amino groups to form stable amide bonds, while (**b**) PEG with thiol-reactive groups is designed for conjugation with thiol groups in cysteine residues. (**c**,**d**) represent PEG-amino acid derivatives, such as PEG-Nle and PEG2-Lys, enabling precise site-specific modifications with amino acid residues. (**e**) PEG succinimidyl carbonate (SC-PEG) facilitates stable covalent attachment through reactions with hydroxyl or amino groups. (**f**,**g**) depict PEG-triazine derivatives, which are highly reactive species for modifying proteins or bioactive molecules via nucleophilic substitution reactions. (**h**) Comb-shaped PEG provides multiple functional sites, enhancing conjugation with higher molecular weight polymers. (**i**) PEG amine is functionalized with amine groups, allowing further chemical modifications. (**j**) Maleimidyl PEG targets thiol groups, forming highly stable thioether bonds, and (**k**) hetero-bifunctional PEG contains two distinct functional groups, enabling dual-site modifications for complex conjugates. These PEG derivatives are extensively used in therapeutic protein conjugation, reducing immunogenicity, extending half-life, and optimizing bioactivity. The figure emphasizes site-specific strategies to enhance therapeutic efficacy [20].

Various PEG derivatives can be used for the pegylation process. These derivatives are usually synthesized using monomethoxy-PEG (mPEG). Some examples of PEG derivatives include (Figure 10):

- Succinimidyl PEG

mPEG with a terminal carboxyl group is a versatile derivative. The carboxyl group can prepare active esters of the polymer and bind it directly to molecules of biological importance. The N-hydroxy succinimidyl (NHS) ester of PEG interacts directly with amino groups in the protein structure between pH 7–9 (Figure 10a,b) [20].

- PEG-succinimidyl carbonate

For PEG activation, the researchers reacted to the hydroxyl group of mPEG with phosgene and activated it with NHS. The resulting structure, PEG-succinimidyl carbonate, reacts with amino groups of proteins between pH 7–10 and forms a durable urethane bond (Figure 10e) [20].

- PEG-amino acid

The mPEG-acid is attached to the α-amino group of norleucine or the ε-amino group of lysine (Figure 10c,d) [20].

- PEG-triazine

2-(O-methoxypolyethyleneglycol)-4,6-dichloro-s-striazine, which is one of the other types of modifiers, as PEG1 (Figure 10f,g) and 2,4-bis (O-methoxypolyethyleneglycol)-6-chloro- s-triazine is abbreviated as PEG2. The primary alcohol in the mPEG structure reacts with cyanuric chloride, replacing one or two triazine groups, respectively, and forming activated PEG1 or PEG2. Activated PEG1 or PEG2 reacts with amino groups of proteins between pH 8–9 and 9.5–10. Two PEG chains are linked to an amino group in the protein structure with a triazine chain, thus providing a more effective protein modification with PEG1 than with PEG2. Such modifying derivatives are called “chain-shaped PEGs” [20].

- Comb-shaped PEGs

It is a PEG and maleic anhydride copolymer and is abbreviated as activated PM. It is in the form of a comb and has multivalent reactive parts. The amino groups of proteins combine with the maleic anhydride in the PM structure to form an amide bond. Comb-shaped PEGs cover the protein molecule and/or place anionic groups (-COOH) on the protein surface (Figure 10h) [20].

- Other PEG derivatives

Apart from amino group modification with PEG derivatives, different strategies have been developed for protein modification. PEG-amine has been used for the modification of carbonyl compounds (Figure 10i). Recently, hetero-functional PEG derivatives have been synthesized as new modifiers (Figure 10k) [20].

#### 2.4.2. Advantages of PEG-Active Substance Conjugates

**Figure 11:**
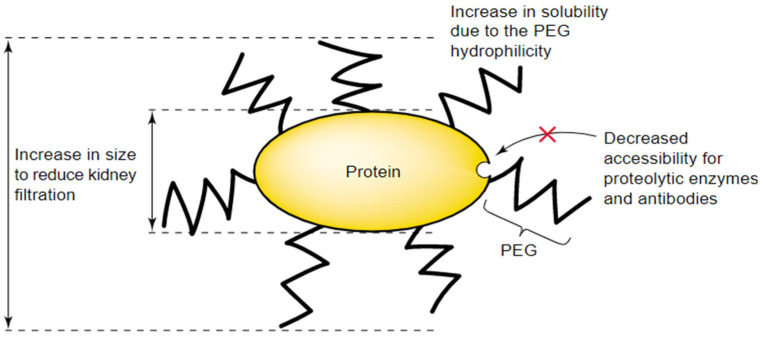
Schematic representation of the advantages of the pegylated protein. This schematic representation highlights the benefits of PEGylation in protein modification. PEG chains reduce immunogenicity, improve solubility, and increase the molecular size to prevent renal filtration. The steric hindrance effect shields the protein from enzymatic degradation, enhancing pharmacokinetic properties [22].

There are numerous benefits that PEGylation conjugation provides, which makes it a preferred method in many biomedical applications (Figure 11). PEG carries its physicochemical properties to the peptide or nonpeptide molecule to which it is attached, thus changing the biodistribution and solubility properties of that substance. The molecular size of the polypeptide is increased, and as a result, its renal ultrafiltration is reduced, as well as increasing the solubility and reducing (or even eliminating) protein immunogenicity [22].

PEG chains also prevent the contact of antibodies or antigen-handling cells and mask the protein surface by steric hindrance, protecting it against degrading agents [22]. In general, the application of PEGylation improves the pharmacokinetic properties of various active substances and renders enzymes and bioactive substances dissolvable in organic solvents or aqueous solutions [20]. PEGylation also shows high efficiency in cancer treatments, as it increases the accumulation in tumor tissues [20].

Functionalization techniques for bioactives, such as PEGylation, have technical-economic-environmental implications. While these technologies enhance therapeutic efficacy, their environmental impact includes energy consumption, waste generation, and potential bioaccumulation of non-biodegradable polymers like PEG. Developing biodegradable alternatives, such as polyglutamic acid or polysialic acid, can mitigate these effects and align with sustainability goals.

#### 2.4.3. Factors Limiting the Use of PEG

PEG is a polydisperse polymer that consists of molecules with different numbers of monomers. The polydispersity of PEG causes some drug conjugates to show disparate biological characteristics of residence time and immunogenicity. Advancements in synthesis and purification methods helped reduce the polydispersity of PEG. However, this complication must be considered, especially when working with peptide or non-peptide low molecular-weight drugs [22].

Another issue related to PEG use is the excretion of the polymer from the body. PEGs are excreted in urine or feces. However, if it has a high molecular weight, it may accumulate in the liver and cause macromolecular syndrome [22].

### 2.5. Site-Specific Conjugation

**Figure 12:**
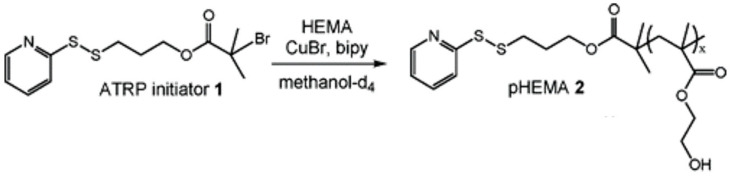
Semitelechelic polymer pHEMA. The figure illustrates a thiol-reactive semitelechelic poly(2-hydroxyethyl methacrylate) (pHEMA) polymer prepared using ATRP. Functionalized disulfides enable site-specific conjugation with free cysteine residues on proteins, bypassing post-polymerization modifications [23].

In addition to PEGylation, researchers developed other methods of protein–polymer conjugation. They aim to devise conjugation techniques that produce bioreactive conjugates with tunable characteristics suitable for various applications. One approach is site-specific conjugation, where free Cysteine residues (i.e., Cysteine residues not involved in disulfide bonds) are targeted for a reaction with semi-telechelic polymers. This type of functional polymer is prepared using sulfhydryl-reactive initiators. One example is the technique developed by Bontempo et al. [23], which can be summarized as follows (Figure 12):

First, the researchers initiated the polymerization of 2-hydroxyethyl methacrylate (HEMA) monomer with pyridyl disulfide-functionalized initiator 1.

The controlled polymerization process was based on the Atom Transfer Radical Polymerization (ATRP) technique, and is mediated by CuBr/2,2′-bipyridine (bipy). This process aimed to functionalize the synthesized polymer with activated disulfides on one end.

The resulting semitelechelic polymer pHEMA is a thiol-reactive polymer with a low polydispersity index [23].

**Figure 13:**
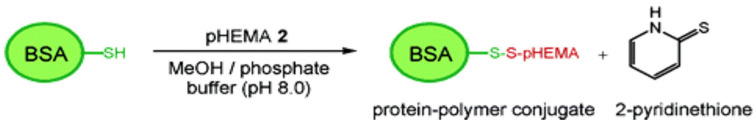
(BSA)-pHEMA conjugation. Bovine serum albumin (BSA) conjugated with pHEMA demonstrates site-specific bioconjugation using thiol-disulfide chemistry. The figure shows incubation and reaction conditions leading to the formation of stable protein–polymer conjugates with preserved protein structure [23].

Then, the researchers successfully conjugated the pHEMA polymer to Bovine Serum Albumin (BSA) protein, which contains free Cysteine residue (Cys-34), by incubating the two materials (at room temperature) for 30 min (Figure 13).

This strategy obviated the need for post-polymerization adjustments in forming protein–polymer conjugates [23].

### 2.6. Protein PEPylation

As a novel advancement in the field of protein–polymer conjugation, this method aims to evade the shortcomings of PEGylation method. It conjugates proteins with synthetic polypeptides (Poly (α-amino acid)s). Synthetic polypeptides have been considered as a promising component for conjugation with proteins for their unique properties:

Synthetic polypeptides are susceptible to chemical and pyrolytic degradation for their peptidic backbone.

Their side chains can be extracted from natural or synthetic origins.

Their ability to form protein-like arrangements (α-helices/β-sheets) by hydrogen bonding allows them to self-assemble into various complex nanostructures.

The following figures illustrate various routes of site-specific protein PEPylation. These approaches intend to produce biorthogonal functionalities on the polypeptide in situ to simplify the synthetic process:

#### 2.6.1. N-Terminal Specific Protein PEPylation via NCL

**Figure 14:**
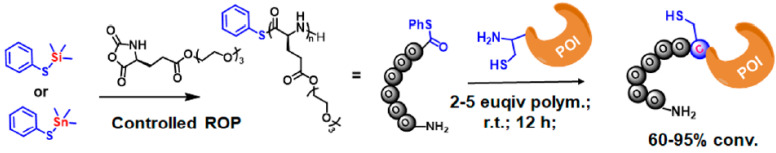
PEPylation of N–terminal specific protein via NCL. The synthesis route for N–terminal-specific protein PEPylation is shown. Reactive thioesters are ligated with engineered proteins containing N–terminal cysteine residues. This technique improves site specificity for synthetic polypeptides in biomedical applications [24].

In this method, Trimethyl Phenylsulfide (PhS-TMS) or Trimetylstannyl Phenylsulfide (PhS-SnMe3) are employed as an initiator for the installation of reactive phenyl thioester on the polypeptide. Then, the resulting polypeptides can form N-terminal specific protein PEPylation when ligated with engineered proteins with N-terminal Cystein (Figure 14).

#### 2.6.2. C-Terminal Protein Conjugation via SML (Sortase A-Mediated Ligation)

Sortase A ligation is a highly specific enzymatic method for protein conjugation. By targeting the LPXTG motif in proteins, it enables site-specific C-terminal ligation without requiring harsh reagents. This method offers advantages over chemical synthesis techniques, including improved reaction specificity, compatibility with sensitive biomolecules, and reduced by-product formation.

**Figure 15:**
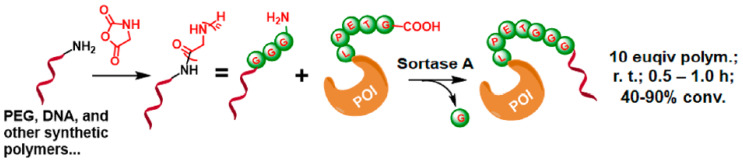
C–terminal protein conjugation via SML. Sortase A–mediated ligation targets the LPXTG motif for precise C–terminal conjugation. The figure depicts the ring-opening polymerization of glycine NCA and the enzymatic ligation to create protein–polymer conjugates suitable for targeted drug delivery [24].

This method depends on the formation of the transpeptidase enzyme Sortase A-mediated ligation. Short aminooligoglycine nucleophile is the substrate for Sortase A and is generated at the end of the polymer by the ring opening Polymerization (ROP) of glycine NCA. Sortase A can act as a mediator for ligating the polymer to proteins containing the LPXTG motif (X = any amino acid) (Figure 15).

#### 2.6.3. Macrocyclic Protein Conjugation via Consecutive NCL and SML

**Figure 16:**
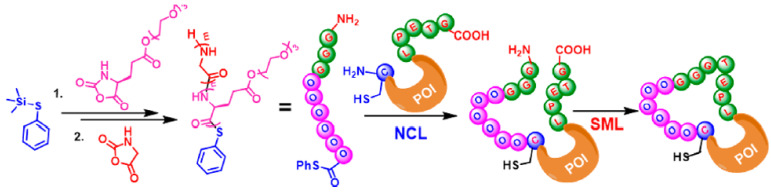
Macrocyclic protein conjugation via consecutive NCL and SML. This diagram combines NCL and Sortase A-mediated ligation to form macrocyclic protein–polypeptide conjugates. The process enables the creation of complex bioconjugates with tailored mechanical and functional properties for biomedical use [24].

Combining the previous two methods can be harnessed to construct PEPylated proteins with complex architecture. As illustrated in Figure 16, the PhSTMS-mediated tandem NCA polymerization can generate a hetero-telechelic polypeptide with a phenyl thioester and an aminoglycine on each end. This complex polymer can undergo successive NCL and SML processes for the PEPylation of an N-terminal Cystein and C-terminal LPXTG motif to construct macrocyclic protein–polypeptide conjugates.

### 2.7. Nanoparticles Formed with Protein–Polymer Conjugates

**Figure 17:**
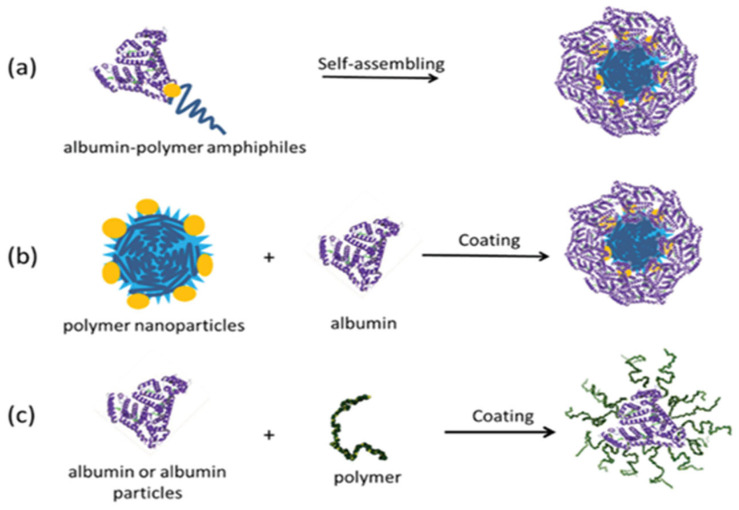
(**a**) Albumin–polymer nanoparticles obtained from large amphiphilic structures, (**b**) polymeric nanoparticles coated with albumin corona, and (**c**) albumin (native or denatured) or albumin particles coated with a polymer. Various nanoparticle structures formed from amphiphilic protein–polymer conjugates are shown. Self-assembled polymer cores and protein coronas enhance drug solubility, stability, and targeted release mechanisms in cancer therapy and other applications [16].

Different structures of nanoparticles can be synthesized using amphiphilic protein–polymer conjugates. As shown in Figure 17, nanoparticles consisting of polymer core and protein corona can be obtained thanks to the self-assembly property of the conjugate obtained by combining the albumin protein and a hydrophobic polymer in the appropriate solvent. Nanoparticles can also be formed where albumin or polymer coats the surface [16].

**Figure 18:**
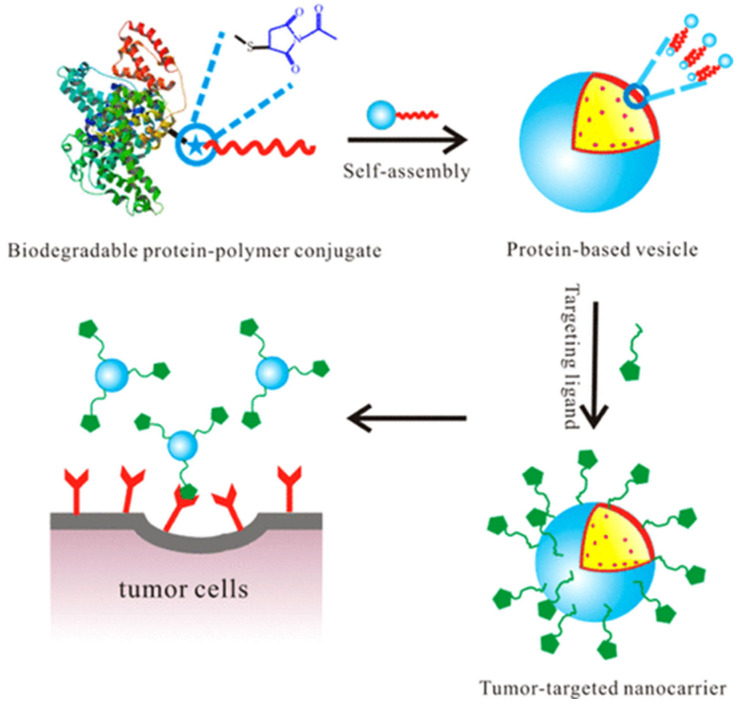
Drug delivery by nanoparticles synthesized from albumin–polymer conjugate. This schematic illustrates the drug delivery process by nanoparticles derived from albumin–polymer conjugates. Ligands target tumor regions, while encapsulated drugs achieve controlled release, improving therapeutic efficacy and reducing side effects [25].

Nanoparticles obtained from protein–polymer conjugates are used as drug delivery systems. In a related study [25], a cancer drug was loaded into nanoparticles obtained by self-assembly of albumin–polymer conjugate to transport the anti-cancer drug to the diseased area more effectively, and its surface was targeted with ligands suitable for the tumor region (as shown in Figure 18).

Nanoparticles synthesized from protein–polymer conjugates act against cancer cells by enhancing drug solubility, protecting drugs from degradation, and achieving controlled release. These nanoparticles, often functionalized with tumor-targeting ligands, exploit enhanced permeability and retention (EPR) effects to accumulate in tumor tissues. Mechanisms include receptor-mediated endocytosis and intracellular drug release, which improve therapeutic efficacy while minimizing off-target effects [26].

### 2.8. Synthesis of Bioconjugates Based on Molecular Recognition

Molecular recognition is a highly effective technique to prepare well-defined polymer–protein conjugates which combine tunable properties of polymers and functionalities of proteins. For that purpose, biotin/streptavidin was widely used in synthesis as biotin binds to streptavidin tetramer with a dissociation constant (Kd) on the order of 10–14 mol/L [27] which is one of the strongest noncovalent interactions known in nature. Many advantages of using molecular recognition in the fabrication of bioconjugates include mild synthetic conditions, high affinity, and high efficiency.

One study conducted by Wang et al. [28] synthesized bioconjugates which were composed of streptavidin and zwitterionic block copolymers of poly (ethylene glycol) (PEG) and poly[3-dimethyl(methacryloyloxyethyl)-ammonium propanesulfonate] (PDMAPS) based on biotin-streptavidin coupling. They were able to fabricate bioconjugates with upper critical solution temperatures (UCSTs) below those of the block copolymers, due to electrostatic screening effect of the protein molecules. Thus, these bioconjugates were able to conduct self-assembly in aqueous solutions at a temperature below UCST. Also, the morphology of the assembled structures is dependent on the average number of block copolymer chains grafted to a protein molecule, thus this paves the way to the synthesis of bioconjugates with unique topological structures.

Another study conducted by Buller et al. [29] synthesized a thermo-sensitive non-ionic copolymer made of oligo (ethylene glycol) methacrylates attached to biotin moieties by free radical copolymerization. The specific binding of streptavidin to these copolymers via biotin induced a marked increase in the lower critical solution temperature (LCST).

Another interesting study conducted by Ding et al. [30] used the thermally responsive polymer poly(N,N-diethylacrylamide) (PDEAAm) that change reversibly from a water-soluble expanded coil to a water-insoluble collapsed globule upon small changes in temperature. They attached it to streptavidin at a site 20 Å away from the binding site of biotin. Below LCST of PDEAAm, the polymer is in its extended state and shields the biotin binding site, thus preventing the biotinylated protein from attaching itself to the polymer. Above LCST, it collapses and exposes the binding site, thereby allowing biotinylated protein binding. This study revealed that the degree of shielding depends on both the size of the biotinylated protein and the size of the polymer. Thus, accordingly, smart polymer shields could be tailored for use in biosensors and diagnostic technologies.

Amphiphilic monodispersed protein-dendron conjugates:

The conjugation of a monodispersed hydrophilic dendron polymer with a hydrophilic protein is one technique used by scientists to create a protein–polymer conjugate that has a polydisperse index of 1.

The hydrophilicity of both the dendron polymer and the protein facilitates the bioconjugation reaction since both components of the conjugate are dissolvable in liquid medium. However, the resultant bioconjugates are not capable of self-assembly because of the absence of hydrophilicity. To overcome this limitation, S. Sandanaraj et al. [31] designed a novel technique for the synthesis of protein-dendron polymers where a hydrophobic dendron polymer is conjugated with a hydrophilic protein to create a facially amphiphilic biohybrid. The type of synthesis technique faces several challenges that complicate the process, including:

1. The diversity of proteins and their functional groups makes the design of site-specific bioconjugation quite complex.
2. Completing bioconjugation reactions in aqueous media is so difficult due to the hydrophobic nature of the dendron-polymer.
3. Conducting chemical analysis or purification processes on this type of hydrophilic bioconjugates is extremely challenging.

S. Sandanaraj et al. [31] group started the bioconjugation process by using cetyl ethylene glycol (CEG) as a monodisperse hydrophilic linker macromolecule. The use of a linker was necessary because the steric hindrance effect and orthogonal solubility properties make a direct and site-specific bioconjugation of a hydrophilic protein to a benzyl ether dendron polymer challenging. The research team chose CEG because its length and flexibility make it possible to avoid steric hindrance. Also, its hydrophilicity and protein-repellent properties protect the conjugated protein from being denatured by it. To achieve this, the researchers employed a slightly modified technique proposed by French et al. to create a monodisperse alkyne-terminated diphosphonate CEG derivative [32].

**Figure 19:**
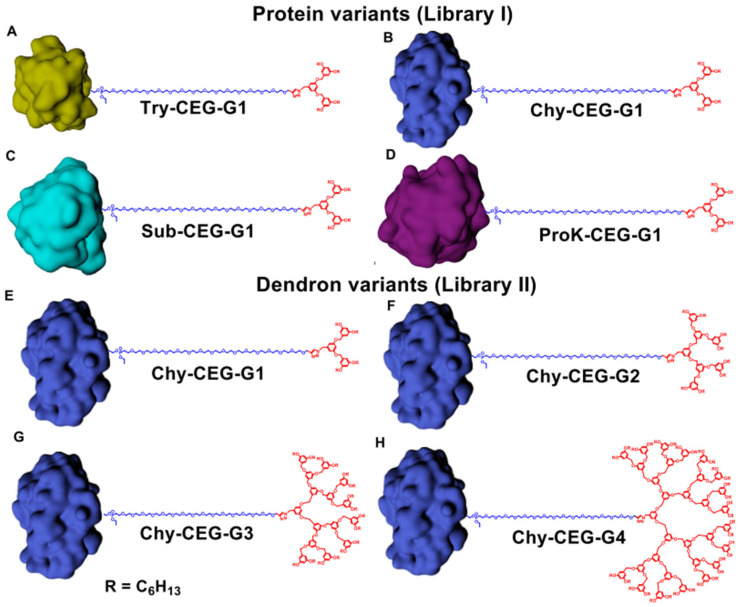
A representation of various types of monodispersed facially amphiphilic protein-dendron bioconjugates (Try: Trypsin, Sub: Subtilisin, Chy: Chymotrypsin, Prok: Proteinase). The diagram shows various facially amphiphilic protein-dendron conjugates synthesized using hydrophilic and hydrophobic components. Structures of (**A**) Try-CEG-G1 (**B**) Chy-CEG-G1 (**C**) Sub-CEG-G1 (**D**) ProK-CEG-G1 (**E**) Chy-CEG-G1 (**F**) Chy-CEG-G2 (**G**) Chy-CEG-G3 (**H**) Chy-CEG-G4. These structures optimize self-assembly and stability for applications in drug delivery and vaccine design [32].

Then, the researchers employed an activity-based protein labeling method for the bioconjugation of an amphiphilic monodisperse macromolecular probe with the protein reactive functional group linked to a hydrophobic dendron polymer. Using this approach protects the structure of proteins from denaturing. Also, they used Frechet-type benzyl ether as the 3D hydrophobic dendron, which was synthesized by a convergent method and contained an azide at the focal point (Figure 19).

The researcher team performed several tests to examine the properties of the synthesized bioconjugates: they applied the SEC studies to measure the ability of the bioconjugates to self-assemble; the DLS experiments in parallel to measure the hydrodynamic diameter (Dh); and the SEC-MALS tests to determine polydispersity, the molecular weight, and the oligomeric state of the protein complexes. The results show that the researchers successfully conjugated a monodispersed facially amphiphilic protein-dendron. Also, the experiments demonstrated that the molecular weight, size, and oligomeric state of this type of conjugate can be controlled by the selection of the protein and dendron polymer. This achievement can have positive implications for biomedical applications, such as targeted drug delivery or vaccine design [32]. Covalently connected protein–polymer nanostructures: this type of amphiphilic micelle consists of a hydrophobic polymer core surrounded by a hydrophilic coronal protein. There are two main categories for core-corona/polymer–protein micelles:

Covalently Connected Micelles (CCMs): the hydrophobic polymer cores and the hydrophilic protein coronae are connected by covalent bonds. They have a stable structure that can endure various tough environmental changes.

**Figure 20:**
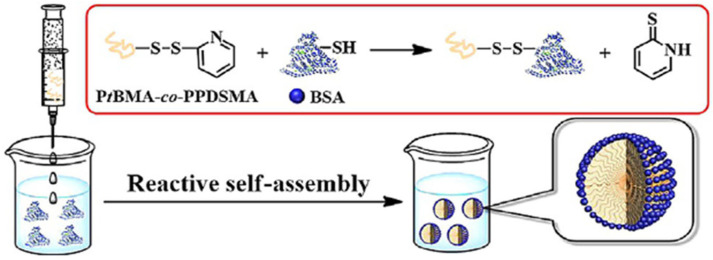
A graph representing the synthesis method of PtBMA134-co-PPDSMA5.5-BSA CCNs micelles. The synthesis of CCMs from BSA and PtBMA-co-PPDSMA is depicted, highlighting disulfide bonding. These micelles provide robust nanocarriers for biomedical applications, maintaining protein integrity and offering size-tunable properties [33].

Noncovalently Connected Micelles (NCCMs): Hydrogen bonds connect the core polymer to the coronal protein. However, hydrogen bonds are fragile and susceptible to changes in external environmental factors like temperature or exposure to salt or solvents, which may lead to the disassociation of the micelle [33]. In their 2017 research paper, Ju et al. suggested a novel technique for the synthesis of polymer–protein micelle nanostructures. The proposed method preserves the structural integrity of the protein and facilitates the reaction between the hydrophilic protein and the hydrophobic polymer. The method depends on the reaction between thiol groups and pyridyl disulfide groups. The synthesis of CCNs micelles begins with the preparation of a hydrophobic polymer with pendant pyridyl disulfide groups; for this purpose, PtBMA-co-PPDSMA polymer was synthesized using reversible addition fragmentation chain transfer (RAFT) polymerization method. Then, the polymer is dissolved into a small amount of water-miscible organic solvent that can be easily removed by dialysis while the hydrophilic protein BSA, which contains cysteine residue, is dissolved in water. Adding PtBMA134-co-PPDSMA5.5 solution to BSA protein solution while stirring causes the aggregation of the polymer chains. After centrifugation, protein molecules covalently connect to polymer aggregates by disulfide bonds due to rapid exchange reaction between the protein’s thiol groups and the polymer’s pyridyl disulfide groups, which also produces pyridine-2-thione (Figure 20). Any precipitated BSA protein molecules that did not participate in the formation of the CCNs micelles are cleared away [33].

The researcher team performed several tests to examine the properties of the synthesized bioconjugates: they applied the SEC studies to measure the ability of the bioconjugates to self- assemble; the DLS experiments are in parallel to measure the hydrodynamic diameter (Dh); and SEC-MALS tests are used to determine the polydispersity, the molecular weight, and the oligomeric state of the protein complexes.

The results show that the researchers successfully conjugated a monodispersed facially amphiphilic protein-dendron. Also, the experiments demonstrated that the molecular weight, size, and oligomeric state of this type of conjugate can be controlled by the selection of the protein and dendron polymer. This achievement can hold positive implications with biomedical applications, such as targeted drug delivery or vaccines design [32].

Core-polymer/coronal-protein nanostructures:

This type of amphiphilic micelle consists of a hydrophobic polymer core surrounded with a hydrophilic coronal protein. There are two main categories for core-corona/polymer–protein micelles:

Covalently Connected Micelles (CCMs): the hydrophobic polymer cores and the hydrophilic protein coronae are connected by covalent bonds. They have a stable structure that can endure various tough environmental changes.

Noncovalently Connected Micelles (NCCMs): Hydrogen bonds connect the core polymer to the coronal protein. However, hydrogen bonds are fragile and susceptible to changes in external environmental factors like temperature, or exposure to salt or solvents, which may lead to the disassociation of the micelle [33].

In their 2017 research paper, Ju et al. [33] suggested a novel technique for the synthesis of the polymer–protein micelle nanostructures. The proposed method preserves the structural integrity of the protein and facilitates the reaction between the hydrophilic protein and hydrophobic polymer. The method depends on the reaction between thiol groups and pyridyl disulfide groups.

One of the factors that influence the size of the formed CCNs micelles is the concentration of its components, such as:

- The increase in the concentration of BSA protein results in the formation of smaller CCNs micelles.
- The increase in the concentration of PtBMA-co-PPDSMA polymer results in the formation of bigger CCNs micelles.

Another factor that influences the size of CCNs micelles is the average number of BSA protein’s thiol groups, where the increase in the average number of thiol groups of BSA leads to the increase of disulfide bonds between the protein and the polymer. This also increases the number of hydrophilic coronal BSA molecules on CCNs’ surfaces, which ultimately results in the formation of smaller sized particles.

Furthermore, the researchers examined the biocompatibility of the CCNs nanostructures by applying CCK-8 assays where the CCNs were incubated with HeLa and COS-7 cells for up to 48 h. The results showed cell viabilities at >90% even with high CCNs concentration (up to 200 μg mL^−1^ concentration).

A different technique for the synthesis of core-polymer/coronal-protein nanostructure was proposed by Wang et al. [34], which can be applied in aqueous media with low catalyst concentration.

This technique depends on the atom transfer radical polymerization (AGET ATRP) system to create a cross-linking activator by electron transfer. Alkyl halide serves as an initiator while a transition metal complex acts as a catalyst. The activator is created by a reducing agent that reduces the transition metal complex’s oxidation state.

In their research, the scientists used the following water-soluble material (Table 3):

BSA was selected as the protein ATRP macroinitiator because of its partially oxidized Cys-34 residue. The BSA protein was modified by thiol disulfide exchange reaction with 2-(2-pyridinyldithio) ethyl 2-bromo-2-methylpropionate. As the synthesis process proceeds and the PHEMA chains are cross-linked, PHEMA-core/BSA-corona nanostructures are generated [34].

The percentage of the BSA protein content also affects the average size of the nanostructure where the increase of the amount of BSA protein leads to the formation of micelles with smaller average sizes. The cytotoxicity tests demonstrated the biocompatibility of the PHEMA/BSA micelles with <90% of cell viabilities and proved to be nontoxic with HepG2 cells (up to 200 μg mL^−1^ concentration).

**Figure 21:**
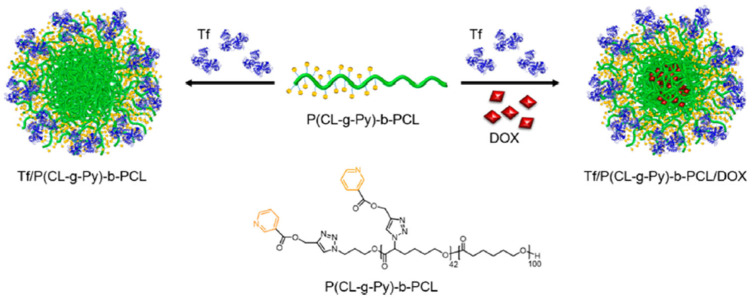
The synthesis of Tf-P(CL-g-Py)-b-PCL CSNPs and the loading of the DOX drug. Human transferrin protein is incorporated into core-shell nanostructures with P(CL-g-Py)-b-PCL for DOX encapsulation. This design targets cancer cells with overexpressed Tf receptors, enabling precise drug release through hydrophobic-hydrophilic interactions [35].

Lu et al. [35] adopted a noncovalent approach for the synthesis of core-shells nanostructure (CSNPs) drug-carriers where they employed pyridine-grafted diblock copolymer instead of homopolymer. In their research, they incorporated the pyridine-grafted diblock copolymer poly(caprolactone-graft-pyridine)-block-poly(caprolactone) [P(CL-g-Py)-b-PCL] with the protein and human plasma transferring (Tf) protein to create a drug carrier structure for the anti-cancer medicine DOX (Figure 21).

The researchers chose human plasma Tf protein for its efficiency as the targeting ligand for cancerous cells that overexpress TfR.

In this diblock copolymer, the P(CL-g-Py) block secures the Tf protein through hydrogen bonds, while the PCL block binds the hydrophobic DOX drug via hydrophobic-hydrophobic interactions. The noncovalent conjugation of the formed core-shell nanostructure preserves the efficiency and structure of the Tf protein.

The synthesis of the diblock copolymer P(CL-g-Py)-b-PCL starts with the sequential open ring polymerization of αClεCL and CL in dry toluene to form P(αClεCL)-b-PCL. Then, the nucleophilic substitution of the chloro groups in P(αClεCL)-b-PCL with azide groups to form P(αN3εCL)-b-CL polymer. Finally, the researchers applied CuAAC coupling to modify the azide group in P(αN3εCL)-b-CL with nicotine propargyl ester (Figure 21).

To load DOX drug, the researchers dissolved the drug with the diblock copolymer P(CL-g-Py)-b-PCL, and then they added the mixture to the Tf protein aqueous solution.

CellTier Blue assay was employed to evaluate the cytotoxicity of the diblock copolymer P(CL-g-Py)-b-PCL using MCF 7 cells and MCF 10A cells. The results demonstrated >90% cell viability, a proof of the superior biocompatibility of the copolymer.

The researchers examined the cellular uptake of Tf-P(CL-g-Py)-b-PCL-DOX bioconjugates using the MCF 7 cells (TfR-positive cells) and MCF 10A cells (TfR-negative cells). The results showed that the bioconjugate had a better targeting of the MCF 7 cells (with an overexpression of TfR) compared to the uptake by the MCF 10A cells (with a regular expression of TfR) [35].

The remarkable properties and biocompatibility of the protein–polymer nanostructures make them an optimal component in many biomedical applications like cancer therapy or drug delivery [33,34,35].

Protein–polymer giant surfactants:

Surfactants are defined as materials that comprise both hydrophobic groups, usually in the form of a long chain, as well as hydrophilic ionic groups.

A giant-size surfactant can be synthesized by the bioconjugation of a hydrophobic polymer chain with hydrophilic protein or enzyme at a specific site.

The synthesis of protein–polymer surfactants has been pioneered using streptavidin (Sav), a homotetrameric protein that can bind biotin noncooperatively in two pairs of oppositely arranged sites, and two molecules of amine-terminated polystyrene bound to the valeric acid carboxyl group of biotins (i.e., monobiotinylated-polystyrene) to create the amphiphilic complex. They applied monolayer techniques to conjugate the two molecules of monobiotinylated polystyrene to streptavidin by spreading the biotinylated polystyrene polymer on the air/water interface in a Langmuir trough, then adding the streptavidin to the subphase. The free sites of streptavidin can bind other biotinylated materials like ferritin, a protein containing iron, or horseradish peroxidase (HRP) [36].

Ding et al. [30] worked with the sav protein/biotinylated-polymer surfactants aiming to experiment with a way to reversibly block protein’s active sites. To achieve this, they attached poly (N, N-diethyl acrylamide) (PDEAAM), a thermally responsive polymer, at short distance (approximately 20 Ǻ) from one of the binding sites of the biotinylated proteins on the streptavidin protein. Depending on the temperature in comparison to the lowest critical solution temperature (LCST) of PDEAAM (i.e., 32 C), we can distinguish between two states:

1. Below LCST: the polymer is extended, and it blocks the conjugation of large biotinylated proteins.
2. Above LCST: the high temperature leads to the collapse of the polymer, and thus it exposes the binding site and permits binding [36,37].

In another experiment, the researchers used reversible addition fragmentation chain transfer (RAFT) polymerization method to create a biotinylated poly (N-isopropylacrylamide) by the hydrolysis of the thioester end and the coupling of a thiol group with a maleimide-functionalized biotin. After the attachment with sav protein, the resultant amphiphilic complexes were used for the capture and release of biomacromolecules in microfluidic devices.

It has also experimented with a different approach for the synthesis of giant amphiphilic molecules where they reduced a disulfide bridge on the outer shell of Candida Antarctica’s lipase B (CALB). This reduction created two free thiol groups for the covalent coupling of a maleimide end-capped polystyrene to a specified position on the surface of the protein. The dispersion of the giant surfactant in water forms micrometer-long fibers that consist of 20–30 nm of micellar rods bundles.

The amine groups of side chains of lysine residues have a high reactivity with nucleophiles, which make them another possible target for the bioconjugation of giant amphiphiles (in addition to the thiol groups of Cysteine), especially because amines are ubiquitous in proteins as each protein holds at least one amine group. However, the possibility of having more than one amine group on the protein results in random conjugation of the polymer, which may affect the bioactivity negatively. To overcome this disadvantage, scientists tried to benefit from the N-terminal α-amine group’s lower pKa. However, even in slightly acidic environments, heterogeneous bioconjugation can still take place.

Click chemistry can also be used in the synthesis of giant surfactant as shown by Maynard et al.’s [23] research. The researchers began their experiment with the use of initiators that contain thioether and disulfide in the ATRP process. Then, they treated the produced polymers with reducing agents like dithio-threitol (DTT) to create mercapto-terminated polymers. Finally, the resultant polymers were conjugated with BSA protein in a thiol group [36].

To improve the technique, they also tried to synthesize a polymer directly from a protein to dodge the challenging coupling of often-incompatible protein and polymer groups. Therefore, they started their experiment by functionalizing biotin with an initiator, and then attaching the functionalized biotin with sav protein. Finally, they employed ATRP method to conjugate the thermal-responsive polymer polyNIPAAm to the sav complex.

This innovative method allows the researchers to determine the number of conjugated polymers and facilitates the purification process of the products.

Another example of the use of click chemistry in the manufacture of giant surfactants was reported, where they used an azide or acetylene for the functionalization of an enzyme. Then, they conjugated the functionalized enzyme to a polymer functionalized with an azide-or-acetylene with a standard click chemistry reaction, which resulted in the formation of giant amphiphilic spherical structures.

Metal-ligand coordination can be used in the manufacture of protein–polymer chain surfactant. One example of this approach starts with using terpyridine ligand to functionalize BSA or CALB. Then, a polymer end-capped with mono(terpyridine) ruthenium (II) complex was added, which resulted in the creation of amphiphiles with various morphologies.

**Figure 22:**
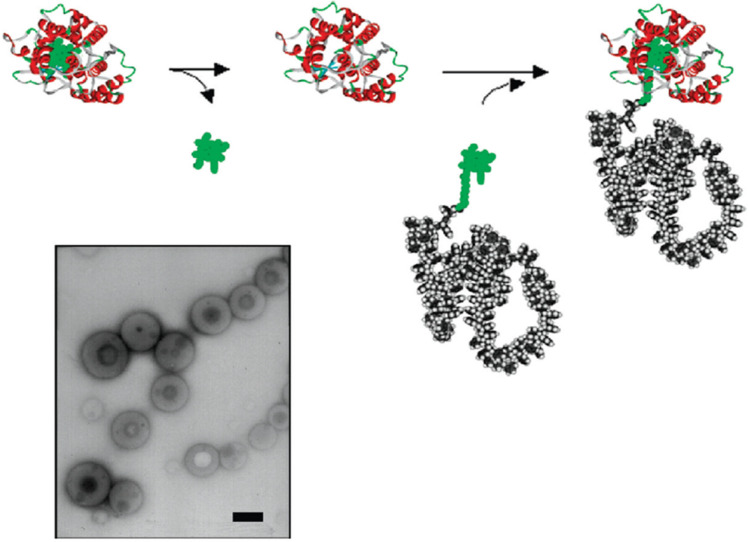
The cofactor reconstitution method for the synthesis of polystyrene-HRP and polystyrene-myoglobin (Mb) biohybrids. The cofactor reconstitution method links polymers to enzyme cofactors before assembly with apoenzymes. The figure illustrates this approach for creating polystyrene-HRP hybrids, enhancing catalytic activity and structural integrity [36].

The cofactor reconstitution method to make polystyrene-HRP and polystyrene-myoglobin (Mb) biohybrids is applied. In this method, the polymer is attached to the cofactor of an enzyme, and then a reconstitution with an apoenzyme follows. Thus, the researchers added a heme cofactor-appended polymer THF solution to an apoenzyme aqueous solution (Figure 22).

This technique resulted in the formation of 80–400-nm-diameter vascular aggregates. The cofactor reconstitution method can also be used for making triblock copolymers in which the block is a protein or an enzyme. Therefore, polystyrene-b-poly (ethylene oxide) diblock copolymer is used for the functionalization of a cofactor using click chemistry with fluorescent zinc derivative that allows the formed copolymers to be characterized with fluorescence spectroscopy [36].

### 2.9. Synthesis Techniques and Tailored Properties of Protein–Polymer Hydrogels

Protein–polymer hydrogels are synthesized using a variety of techniques, including free radical polymerization, Michael addition, enzymatic crosslinking, click chemistry, and ionic gelation. For instance, GelMA (gelatin methacrylate) hydrogels are formed by methacrylation of gelatin, followed by UV-induced crosslinking in the presence of a photoinitiator, allowing precise control over crosslinking density. Similarly, silk fibroin-polyethylene glycol hydrogels are synthesized through enzymatic crosslinking using horseradish peroxidase and hydrogen peroxide, enabling biocompatibility and mild reaction conditions. Another example is hyaluronic acid-tyramine hydrogels, which are formed through peroxidase-mediated oxidative coupling, creating scaffolds with tunable degradation rates suitable for tissue engineering applications.

The physical properties of protein–polymer hydrogels are highly customizable and depend on the synthesis method, polymer type, and crosslinking strategy. Mechanical properties, such as stiffness and elasticity, can range from 1 kPa for soft hydrogels used in neural tissue engineering to over 100 kPa for load-bearing applications such as cartilage regeneration. Porosity, often in the range of 50–200 μm, is crucial for nutrient diffusion, oxygen transport, and cellular infiltration, with highly porous hydrogels favoring tissue regeneration. Swelling behavior, controlled by the hydrophilicity of the polymer and crosslinking density, dictates water uptake and influences the kinetics of drug release. For example, dextran-based hydrogels exhibit high swelling ratios, making them ideal for controlled drug delivery, while collagen-based hydrogels provide a balance of mechanical integrity and hydration suitable for wound healing.

Thermal and pH responsiveness are additional physical properties engineered into certain protein–polymer hydrogels. For instance, amphiphilic protein–polymer conjugates like poly(N-isopropylacrylamide) (PNIPAM) hydrogels exhibit thermo-responsive behavior, transitioning from a sol to a gel state near physiological temperatures. pH-sensitive hydrogels, such as chitosan-based systems, respond to acidic or basic environments, enabling site-specific drug release in applications like cancer therapy. By combining these properties with degradability, hydrogels can be tailored for diverse biomedical applications, ranging from injectable scaffolds and wound dressings to advanced drug delivery systems.

### 2.10. Importance of Molecular Weight in Protein–Polymer Conjugates

The molecular weight of polymer components is a critical determinant of the physical, chemical, and biological properties of protein–polymer conjugates. For instance, polyethylene glycol (PEG) is commonly used with molecular weights ranging from 2 kDa to 20 kDa, where lower molecular weights (e.g., 2–5 kDa) facilitate rapid renal clearance and are suitable for imaging agents, while higher molecular weights (e.g., 10–20 kDa) provide enhanced stability and extended circulation times, ideal for therapeutic protein conjugation. PEGs exceeding 40 kDa are sometimes used for drug delivery formulations requiring extremely prolonged half-lives, though they may compromise cell permeability and clearance rates.

In amphiphilic block copolymers, the molecular weight ratio between hydrophilic and hydrophobic blocks determines their self-assembly behavior. For example, poly(ethylene oxide)-poly(propylene oxide) (PEO-PPO) block copolymers with a PEO molecular weight of 5 kDa and a PPO molecular weight of 2–3 kDa form stable micelles, making them effective as solubilizers for hydrophobic drugs. Conversely, PEO-PPO copolymers with a PPO molecular weight exceeding 4 kDa shift toward vesicle formation, which is advantageous for encapsulating larger drug payloads.

Polymers like dextran and hyaluronic acid also exhibit molecular weight-dependent behaviors. Dextran with molecular weights of 10–40 kDa is often conjugated with proteins for enhancing drug solubility and circulation, while dextran exceeding 70 kDa significantly improves mechanical strength in hydrogel formulations but may hinder biodegradation. Similarly, hyaluronic acid with molecular weights of 1–3 MDa (megadaltons) provides superior viscoelasticity and hydration, essential for cartilage regeneration and dermal fillers. However, lower molecular weights, such as 50–100 kDa, are preferred for drug delivery applications requiring rapid degradation and systemic clearance.

Another example is polyglutamic acid (PGA), where molecular weights of 10–30 kDa are used for drug conjugation to improve stability and control release rates, while molecular weights exceeding 50 kDa enhance mechanical integrity in hydrogel networks. Similarly, in poly(lactic-co-glycolic acid) (PLGA), molecular weights between 10–50 kDa are optimal for controlled release formulations, balancing degradation rates and structural stability.

Understanding molecular weight distribution and polydispersity is crucial for ensuring reproducibility and optimizing performance. Polydisperse systems, such as those with a wide range of molecular weights, can result in inconsistent drug release profiles or mechanical properties. Techniques like gel permeation chromatography (GPC) and matrix-assisted laser desorption/ionization mass spectrometry (MALDI-MS) are essential for accurate characterization. By precisely tailoring molecular weights, protein–polymer conjugates can be optimized for specific biomedical applications, such as drug delivery, tissue engineering, and injectable scaffolds.

## 3. Biodegradability

The polymer–protein conjugates have different degradability according to their polymer type. The most common polymer used in polymer–protein conjugates is poly (ethylene glycol) (PEG) [26]. However, PEG is a non-biodegradable polymer, which may cause accumulation in the body. For this reason, biodegradable alternatives to PEG have been researched for protein–polymer conjugates [38].

**Figure 23:**
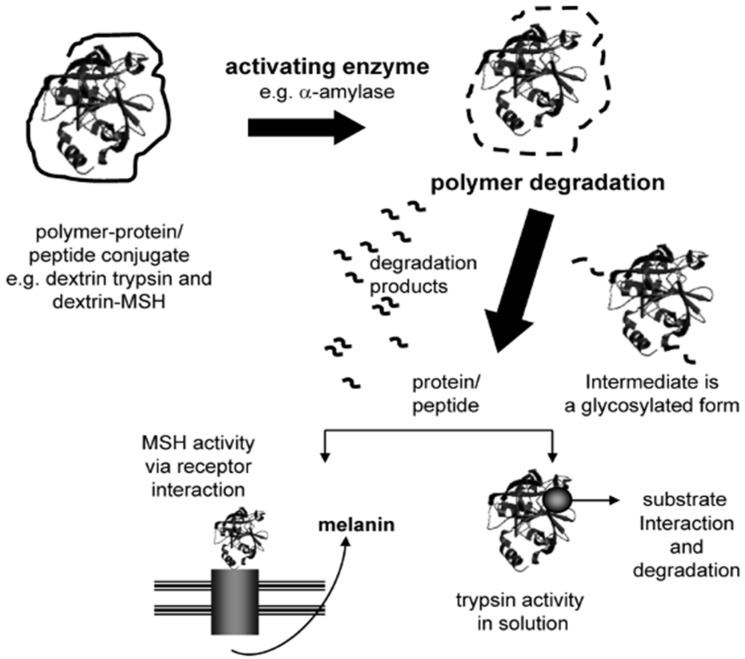
PUMPT concept. The PUMPT concept uses degradable polymers to mask proteins during transit, unmasking them at the target site. Triggered degradation by enzymes or pH conditions reinstates protein activity, providing controlled therapeutic release for wound healing and cancer therapy [39].

One of the alternatives is Polymer Masked–UnMasked Protein Therapy (PUMPT), presented by Duncan et al. PUMPT aims to conjugate the protein to a biodegradable polymer to protect (mask) the protein in transit with the polymer while using the degradation of the polymer to control the reinstatement of activity (Figure 23). The polymer degradation is triggered at the activation site, leaving the protein unmasked. This concept was tested using dextrin–trypsin conjugate (degradable by α-amylase) for wound healing and as an anti-cancer agent, which showed promising results [39].

After the positive outcomes of PUMPT studies with dextrin-α-amylase, other polymers were also tested. One of these polymers is hyaluronic acid- epidermal growth factor (EGF) conjugate was tested for tissue repair; however, the outcome suggested further studies [40].

A study on the PUMPT system triggered by pH has been conducted using biodegradable polyacetals (Pas) and trypsin by Escalona et al. Using the acidic pH caused by disease as a trigger, they successfully created a PUMPT system that masks the protein at p 7.4 and unmasks acidic conditions [41].

Another polymer tested for PUMPT systems is poly (L-glutamic acid) (PGA), which is biodegradable by the lysosomal enzyme cathepsin B. PGA-protein conjugates (with protein lysozyme) have been tested for tumor treatment and showed promising results [42]. Polysialic acid (PSA)-protein conjugate is another biodegradable alternative and has been investigated and shown potential as a drug carrier [43].

Non-degradable polymer–protein conjugates, such as those incorporating polyethylene glycol (PEG), present unique challenges in biomedical applications due to their persistence in biological systems and the environment. Techniques like enzymatic degradation, hydrolysis, and photodegradation offer potential solutions for managing these materials. Enzymatic degradation involves the use of specific enzymes, such as proteases or esterases, to selectively cleave polymer bonds, enabling controlled breakdown into smaller, more manageable fragments. Hydrolysis relies on chemical reactions with water under controlled pH and temperature conditions to achieve degradation, often targeting ester or amide bonds within the polymer structure. Photodegradation utilizes light energy, typically ultraviolet (UV) radiation, to induce bond cleavage in photosensitive polymers. This method is particularly effective for materials that incorporate photo-reactive groups.

Despite these strategies, non-degradable conjugates, such as PEGylated materials, may persist in vivo, leading to potential bioaccumulation and long-term toxicity risks. Persistent polymers can interfere with normal metabolic pathways and accumulate in organs such as the liver and kidneys, potentially causing adverse effects. Furthermore, their presence in the environment raises ecological concerns, as they may resist natural degradation processes and contribute to pollution. To mitigate these risks, the development of biodegradable alternatives, such as polyglutamic acid (PGA) or polysialic acid-based conjugates, is a promising approach. Additionally, regulatory frameworks should encourage the adoption of environmentally friendly materials and support research into effective degradation methods. Careful consideration of the lifecycle of polymer–protein conjugates, from synthesis to degradation, is essential to minimizing their biological and environmental impact.

## 4. Cytotoxicity

The polymer–protein conjugates have different degradability according to their polymer type. The most common cytotoxicity refers to the ability of a substance to cause harm or death to cells. It is essential in developing polymer–protein conjugates, hybrid molecules composed of a polymer chain covalently linked to a protein. These materials have many potential applications in the pharmaceutical, biomedical, and biotechnological industries. Still, their success depends on their ability to effectively deliver their protein payloads to specific sites in the body without causing harm to healthy cells [44].

Several factors can influence the cytotoxicity of polymer–protein conjugates. Table 4 summarizes them. One crucial factor is the polymer itself. Some polymers are cytotoxic, either because they have chemical groups that are toxic to cells or because they interfere with cell function through mechanisms such as physical entrapment or aggregation [45]. On the other hand, other polymers are biocompatible and non-toxic, making them suitable for polymer–protein conjugates [46]. Another factor that can impact the cytotoxicity of polymer–protein conjugates is the protein itself. Some proteins are inherently cytotoxic because they are toxic to cells or stimulate an immune response that leads to cell death [47]. It is essential to carefully consider the choice of protein when designing polymer–protein conjugates, as the protein can significantly affect the overall cytotoxicity of the material.

The method of conjugation can also influence the cytotoxicity of polymer–protein conjugates. Conjugation methods involving chemical crosslinking or toxic reagents can introduce cytotoxic groups into the material, leading to increased cytotoxicity [48]. On the other hand, methods that rely on more gentle chemical reactions or physical entrapment can reduce cytotoxicity [49]. The size and shape of polymer–protein conjugates can also impact their cytotoxicity. Larger conjugates tend to be more cytotoxic than smaller ones, likely due to their increased ability to physically entrap or aggregate cells [50]. Similarly, conjugates with highly irregular shapes may be more cytotoxic due to their ability to disrupt cell function through physical interference [45,51].

Finally, the delivery method can also affect the cytotoxicity of polymer–protein conjugates. Some delivery methods, such as intravenous injections, might be more cytotoxic than others due to the stresses of injection or the presence of foreign substances in the delivery vehicle [52].

In summary, cytotoxicity is an essential consideration in designing and developing polymer–protein conjugates. Several factors can influence the cytotoxicity of these materials, including the polymer, protein, conjugation method, size and shape, and delivery method. It is essential to carefully consider these factors to maximize the effectiveness and safety of polymer–protein conjugates.

## 5. Applications

### 5.1. Biomedical Applications

New biomedical applications of proteins as treatments for various illnesses have been introduced over the past decade. They can be effective therapeutics in conditions that are the result of the deficiency or reduced activity of specific proteins or potent inhibitors of biological processes. However, if proteins are exposed to external environmental changes, like pH and temperature, they lose their stability or may even denature with time. In addition, proteins have intrinsic properties like short half-life, low in vivo stability, and immunogenicity that limits their efficiency in biomedical applications. Consequently, a high protein dose is needed to achieve a significant effect in vivo, which could be toxic to healthy organs and tissues and may lead to many side effects. Conjugation of polymer chains with proteins decreases the renal excretion rate and increases the hydrodynamic volume. The side chain can protect the protein from enzymatic degradation and improve the serum half-life. PEGylation is one highly powerful conjugation method for the enhancement of the pharmacokinetic properties of the protein [53,54].

Previously, in bioconjugate therapeutics, PEGylation was ubiquitous, improving pharmacokinetics and reducing immunogenicity by altering the physiological properties of the molecules [55,56]. However, new bioconjugates, such as “smart polymer”, are being introduced. These new types of bioconjugate have multiple applications in drug delivery and sensing [57].

This section will illustrate an overview of various biomedical applications of protein–polymer conjugations.

#### 5.1.1. Drug Delivery

In their development of protein–polymer conjugate drug carriers, researchers aim for a design with high biocompatibility, biodegradability, as well as low toxicity and optimize the carrier’s structure to achieve characteristic enhancements, such as:

- Temporal control: which is the drug release as a response to an external stimulus (e.g., heat, light, etc.).
- Distribution control: which is the targeted delivery of the drug [2].

Conjugation meets optimal therapeutic needs like the enhancement of solubility and the prevention of phagocytosis of therapeutics [58] as well as the extension of its stability in the bloodstream [59,60] by synergistically producing hybrid materials that combine the properties of its individual components [58].

Researchers in this field use various conjugation methods like reversible deactivation radical polymerization (RDRP) and ROMP for the synthesis of protein–polymer conjugates [58].

Responsive Protein–polymer Conjugates:

Employing a responsive polymer in the design of the self-assembly conjugates helps with temporal control, where the drug is released as a response to the external stimulus [2,58]. These polymers are a wide range of compounds that can change color and shape in response to external stimuli like pH, humidity, and light variations. Proteins like enzymes have been used by molecular collapse or expansion of the smart polymer chain when subjected to the stimuli mentioned earlier, which cause steric blocking and unblocking of the protein’s active site. Responsive (Smart) protein–polymer conjugates are used in drug delivery where they can control drug release. It can also be used in protein purification [55,61].

Vanparijs et al. [62] employed this remarkable aspect of conjugation to design a transiently responsive conjugate to secure the discharge of the polymer. A transiently responsive conjugate is a soluble thermo-sensitive conjugate that responds to a temperature stimulus by aggregation. However, acidic hydrolysis will cause the conjugate to remain soluble regardless of temperature. Thus, the conjugate preserves its solubility within the acidic endosomal interior and dodges excess aggregation of the conjugate.

In their experiment, researchers used [(2,2-dimethyl-1,3 dioxolane methyl)] acrylamide (DMDOMA), a thermo-sensitive polymer synthesized by Kizbakkedathu et al. [63]. The hydrolysis of the DMDOMA’s dioxolane groups into diols raises the polymer’s temperature TCP until it gains complete solubility regardless of temperature. Using a grafting technique and reversible addition-fragmentation chain transfer (RAFT), Vanparijs et al. [62] conjugated a pDMDOMA polymer to Bovine Albumin Serum (BAS) protein. After increasing the temperature above the lower critical solution temperature (LCST), the polymer will self-assemble into a nanoparticle that can serve as an intracellular carrier of protein and hydrophobic materials due to its amphiphilicity of the conjugate, as shown by the experiment of the successful delivery of the hydrophobic CL075 molecule into dendritic cells [62].

Wong et al. used the more responsive polymer poly(N-isopropylacrylamide) (or PNIPAM) to conjugate to coral reef species (Acropora Millepora) amilFP497 protein. An increase in temperature above LCST transforms the PNIPAM into a hydrophobic polymer. At 37 C, the thermo-responsive PNIPAM-b-amilFP497 block copolymer conjugate forms polymersomes. These polymersomes encapsulated the hydrophilic anti-tumor drug DOX in its core and membrane [64].

Another example of a responsive bioconjugate is Ji et al.’s conjugation of thiol-modified antibacterial protein lysozyme with thermal-responsive polymer poly (DEGMA250-co-PDSMA12). Lysozyme’s antibacterial effect depends on the catalysis of the hydrolysis β (1–4) bonds between N-acetyl-d-glucosamine and N-acetylmuramic residues in peptidoglycans, which are the components that make up 90% of cell walls in gram-positive bacteria. The antibacterial efficiency of the lysozyme protein makes it the subject of research that tries to secure lysozyme with a polymer to assemble a reusable conjugate that can be used in the food packaging industry [65].

For their research, Ji et al. [65] modified lysozyme with Traut’s solution (2-iminothiolane) to make thiol-modified lysozyme with one thiol group. The (DEGMA250-co-PDSMA12) copolymer was synthesized with the reversible addition-fragmentation chain transfer (RAFT) technique where the researchers assembled di (ethylene glycol) methyl ether methacrylate (DEGMA) with 2-(2-pyridyldisulfide) ethyl methacrylate (PDSMA). Finally, the conjugation of thiol-modified lysozyme with (DEGMA250-co-PDSMA12) copolymer through thiol-disulfide exchange reaction between thiols and pyridyl disulfide groups, which created pyridine-2-thione. The resultant bioconjugate is thermal-responsive and comprises pendant lysozyme molecules with preserved secondary structure and bioactivity.

As a thermal-responsive structure, the resultant bioconjugate reacts to thermal changes. In aqueous media at temperatures below cloud point (Tcp) temperatures, the bioconjugate creates core-shell nanostructure as a result of the unfavorable interaction between the polymer chains and enzyme molecules. While the interchain associations of PDEGMA chains form a mesoglobular structure of the bioconjugate at temperatures above Tcp. The experiments’ results show that Tcp is directly proportional to the number of pendant lysozyme molecules while it’s inversely proportional to salt concentration [65].

Heredia et al. [66] modified free cysteine residue of BSA and a mutant “V131C” form of T4 lysozyme holding a free cysteine with a disulfide or S-C-linked initiator to achieve in situ bioconjugation of initiator moieties to free thiol residues on protein and complete the polymerization from protein macroinitiator of (N-isopropylacrylamide) (NIPAAm) to form the thermally responsive polymer poly (NIPAAm). This approach helps scientists use mass spectroscopy techniques to determine the number and locations of initiation sites and facilitates the purification process of small unreacted molecules by dialysis or chromatography [65].

The researchers modified BSA protein with a 2-bromoisobutyrate group which is a common ATRP initiator. Following the modification of BSA protein, the polymerization of NIPAAm from the BSA macroinitiator takes place in water at ambient temperatures. In the same manner, the polymerization of NIPAAm from the modified lysozyme was also completed with the addition of functionalized resin in water at an ambient temperature. After 90 min to block the polymerization process, the mixture was exposed to air and a centrifugation process was applied to remove the resin. The resultant lysozyme-poly NIPAAm conjugate preserved the same bioactivity of the unmodified enzyme [66].

Albumin-Based Protein–Polymer Conjugates:

Albumin is the most abundant plasma protein, and it is widely used in the synthesis of drug-delivery protein–polymer conjugates due to its quality properties of biodegradability and reduced immunogenicity as the numerous hydrophobic cores that enable it to serve as a transporter of hydrophobic molecules [67]. Researchers employed both Human Serum Albumin (HSA) and Bovine Serum Albumin (BSA) for drug delivery applications (Table 5) [16].

Yang et al. proved Human Serum Albumin (HSA) to be a superior alternative to the conventional PEGylation method for prolonging a therapeutic by conjugating it to Superfolder Green Fluorescent protein (sfGFP). HSA exhibited the same half-life extension efficiency of PEG molecules with less immunogenicity and better degradability [68].

One example is Soo Gil et al.’s experiment to resolve the brevity of the protein’s half-life. Fixing this critical issue is essential for improving the treatment procedure as it decreases the required doses. They exploited HSA abilities to construct injectable hydrogel for drug delivery. They conjugated the HSA to the pH and temperature-sensitive polymer poly (β-aminoester Urethane) and tested the resulting conjugate ability to transport the Hyperuricemia-diseases treatment uricase (Uox). HSA-poly (B-aminoester Urethane) conjugate hydrogels prevented rabid blood expulsion of Uox molecules and enhanced the overall efficiency of the drug in mice with hyperuricemia [69].

The thiolation of HSA using the mild thiolation agent 2-IT is an effective method for the thiolation of HSA without denaturing the protein’s structure, as was shown by Gao et al. They used the thiolated HSA (sHSA) for conjugation with the maleimide functionalized polymer Dextran (Dex-Mal). The resulting in situ gelated DEX-sHSA hydrogels can transport hydrophobic therapeutics. The mechanical properties of these hydrogels can be controlled by varying the concentration and the thiolation degree of sHSA proteins [70]. However, at 37 C, DEX-sHSA hydrogels can only survive degradation for one week. Therefore, the researcher employed poly (ethylene glycol) to extend the viability of the hydrogel up to several weeks at 37 C. They applied a thiol-vinyl sulfone Michael addition reaction to assemble an improved hybrid comprising Vinyl sulfone-modified dextran, Poly (ethylene glycol), and sHSA. The developed DEX (VS)-sHSA-PEG hydrogels were proven to be an efficient transporter of the anti-cancer drug DOX [71].

Thiolated HSA (sHSA) was conjugated with 4-arm PEG-maleimide to produce an injectable PEG-cross-linked albumin hydrogel. The hydrogel was designed and employed for the targeted delivery of the anti-cancer TRAIL (TNF-related apoptosis-inducing ligand) proteins by Kim et al. The HSA-SH/PEG-MAL hydrogel formed in situ within 60 s in optimal conditions and exhibited an outstanding anti-tumor efficiency [72].

Albumin-based hydrogels can also be used for the encapsulation and delivery of vaccines. As in Phan et al.’s synthesis of a hybrid hydrogel composed of the conjugation of triblock copolymer (PCLA) to Bovine Serum Albumin (BSA) in a three-step process as a mean for plasmid DNA (pDNA) vaccine encapsulation and delivery. This hydrogel proved an excellent recruiter for dendritic cells, an Antigen-Presenting Cell (APC). The researchers noted that merely one dose of the pDNA vaccine containing BSA-PCLA hydrogel was sufficient to develop an Aβ-specific humoral immunological memory [73].

Jiang et al. provided a simple and cheap method for forming albumin-based nanocarriers. Using maleimide-terminated poly (methyl methacrylate) PMMA, they synthesized a self-assembled carrier of the anti-cancer drug curcumin. Three simultaneous processes can occur during preparation (conjugation, self-assembly, encapsulation). Thus, this one-pot approach for the manufacture of albumin-based nanocarriers benefits from the specificity of albumin to target cancerous cells [74].

These were a few examples of other albumin-based conjugates, which is a testament to the high efficiency of albumin-based conjugates as a drug carrier.

Delivery to Difficult Targets:

An optimal drug carrier should secure targeted drug delivery. Targeting tumor cells accurately reduces the negative side effects of cancer therapy. Also, infiltrating the blood-brain barrier holds hope for delivering therapeutic materials for neurological diseases like Alzheimer’s.

**Figure 24:**
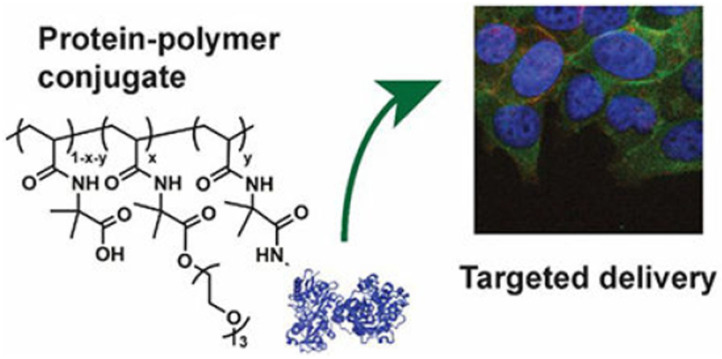
A representation of targeted delivery. This figure demonstrates the structure and function of a responsive protein–polymer conjugate that undergoes a conformational change in response to temperature or pH. The smart polymer component controls drug release by expanding or collapsing, providing tunable delivery in response to external stimuli, enhancing precision in therapeutic applications [75].

One approach to surmount such challenges is the synthesis of hTF-PVMDA-mTEG conjugates. In this conjugate, poly (2-Vinyl-4,4-dimethylazlactone) PVDMA polymer functionalized with triethylene glycol monomethyl ether (mTEG) to transform it into a hydrophilic polymer. Then, the functionalized polymer conjugated with holo-transferrin (hTF) protein. The hTF-PVMDA-mTEG conjugates showed high efficiency in targeting cells with transferrin receptors (Figure 24). This method paves the way for further advancement in targeted drug delivery to overcome challenges related to the treatment of tumors and neurological diseases [75].

#### 5.1.2. Tissue Engineering

Another field where scientists benefit from the superior characteristics of conjugates is tissue engineering. This critical field of regenerative medicine witnessed notable advancements thanks to the adoption of protein–polymer conjugates. Laboratories worldwide strive to develop prime biomaterials that can act as a scaffold for regenerating tissues.

One example is the Extracellular Matrix (ECM) proteins, one of the most ubiquitous biomaterials used in tissue engineering applications. ECM proteins can construct 3D hydrogel matrices and biological scaffolds. Therefore, researchers work to improve the control of physical properties and prevent the quick degradability of the ECM proteins. One approach to achieving these goals was completed by Gonen-Wadmany et al., where they constructed a matrix composed of three PEGylated ECM proteins (collagen, fibrinogen, albumin) with free radical photopolymerization. The experiment results showed that the PEGylation of the ECM proteins provided improved control of the structural properties and biodegradability of the PEGylated proteins while preserving cell adhesion, bioactivity, and biocompatibility [76].

Also, protein–polymer conjugates showed remarkable performance in wound healing, as was demonstrated by Phan et al. where they conjugated triblock polymer PCLA to BSA protein for the in situ formation of a biocompatible injectable gel at physiological temperature (Figure 21). This gel can accelerate the recovery process of the wound without causing any immunological response [77].

**Figure 25:**
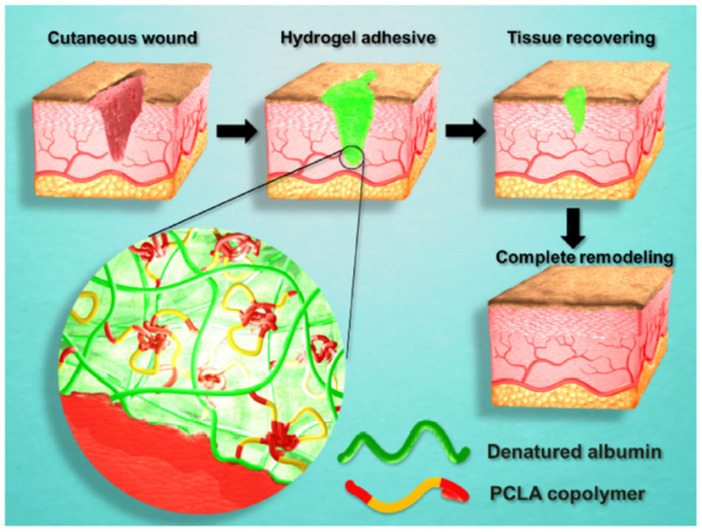
Wound healing with BSA-PCLA bioconjugate. The schematic shows how protein–polymer conjugates are integrated into hydrogel matrices. Crosslinked networks of conjugates mimic extracellular matrix structures, offering mechanical strength, biodegradability, and controlled porosity. Applications include tissue engineering scaffolds and controlled drug delivery systems [79].

Hyaluronic acid-based protein–polymer conjugates have been employed for cartilage regeneration since hyaluronic acid composes the ECM of cartilage [4]. Zhu et al. synthesized an elastin-like protein (ELP) and hyaluronic acid (HA) hydrogel conjugate to form a matrix for cartilage tissue engineering that allows the increase of HA concentration without the rise of its mechanical stiffness (Figure 25) [78].

#### 5.1.3. Anti-Cancer/Cancer Therapy Applications

Conjugated polymer proteins are used for enhanced cancer therapy applications for high-accuracy tumor killing and minimal invasiveness. In sonodynamic therapy (SDT), reactive oxygen species (ROS)-responsive nanoscale coordination polymers (NCPs), which contained ROS-cleavable thioketal (TK) linkers, were designed through the self-assembly of porphyrins (PP) and platinum to improve the delivery of doxorubicin (Dox). Based on that, the Dox-loaded NCPs (PTK@PEG/Dox) may produce a significant amount of cytotoxic ROS and heat while subjected to ultrasound (US), attaining the cooperative therapy of US-induced therapy and chemotherapy [80].

In another article, the anti-cancer polymers were made to damage the membranes of cancer cells by killing dormant docetaxel-resistant tumor cells and showing cytotoxicity in multiplying prostate cancer. Moreover, polyacrylic acid-b-polyaniline (PANI-b-PAA), a charged amphiphilic block copolymer with anti-cancer and antibacterial features, is appropriate and more effective for colorectal cancer treatment as drugs for this third most common cancer are harmful to healthy cells [81].

Another similar research is about a novel copolymer beneficial in prostate cancer (PC) treatment. The copolymer was inspired by anti-cancer peptides (ACPs). ACP-mimetic polymers specifically target PC cells and kill dormant PC cells resistant to anti-cancer medications. Additionally, they can be chemically tuned for activity modulation more than peptides, and they are more resistant to proteolytic degradation in physiological settings [82]. An additional related polymer is anti-cancer heterochiral β-peptide polymers as synthetic imitates of host defense peptides to fight against multidrug-resistant cancers [83].

Another application is drug loading. Usually, the drug Tamoxifen which has challenges like poor bioavailability and low hydrophilicity, is prescribed for breast cancer, the second most common cause of cancer-related deaths in women. Therefore, it was aimed at creating chitosan- poly (lactic-co-glycolic) acid (PLGA) copolymer micelles that were loaded with tamoxifen to administer this promising drug more safely and effectively [84].

To deliver anti-cancer medications that target tumor cells, a unique dual-pH sensitive charge-reversal approach is developed because it is found that the tumor extracellular natural environment is more acidic with a pH of 6.2–6.9 than blood and normal tissues that are of pH 7.3–7.4. The lysine amino acids are amidated by 2,3-dimethylmaleic anhydride to form carboxylic amide, which causes the polypeptides to self-assemble into negatively charged micelles and poly(L-lysine)-block-poly(L-leucine) diblock copolymer is created [85]. The advanced polymeric application for cancer therapy has shown a wide range of improvements in this field.

The structure of protein–polymer hydrogels is intrinsically linked to their function in biomedical applications. For instance, the crosslinking density directly impacts the stiffness and elasticity of hydrogels, with higher crosslinking resulting in increased mechanical strength but reduced swelling and nutrient diffusion. Porosity plays a critical role in tissue engineering, where interconnected pores promote cell infiltration and vascularization. In drug delivery applications, the hydrogel’s degradation rate, dictated by the polymer backbone’s susceptibility to hydrolysis or enzymatic cleavage, determines the release profile of encapsulated drugs. For example, hydrogels with dynamic covalent bonds enable stimuli-responsive behavior, such as pH- or temperature-triggered drug release, making them ideal for site-specific therapeutic applications.

### 5.2. Miscellaneous Applications

Polymer-based protein engineering has emerged as a powerful tool for protein scientists to improve enzyme properties and facilitate their use in industrial enzyme applications. To broaden the application of proteins for industrial applications beyond the medical field, research has been focused on methods to generate stable, selective, and productive proteins and enzymes, which can accept a variety of substrates and transform them into novel materials, high-value chemicals, and renewable biofuels. It has been shown that the covalent attachment of a water-soluble polymer to a protein improves physical stability, proteolytic stability, and pharmacokinetics in therapeutic applications. The same approach has been applied in biocatalysts, where polymer-grafted enzymes displayed increased solution and thermal stability and improved performance in nonaqueous solvents [86]. Such enzyme-polymer-based biocatalysts can be promising tools for active pharmaceutical ingredients synthesis, commodity and specialty chemicals synthesis, waste remediation, coatings, and packaging applications.

Applying protein–polymer conjugates includes specialty areas such as fuel cells, sensors, and coatings. The application of protein–polymer conjugates in coatings is another promising area due to the interest in biocatalytic coatings for synthetic applications, sensors, or innovative packaging. Such enzyme-containing coatings can be produced by flow coating of enzyme-polymer conjugates. One can utilize self-assembly processes to generate 5–10 times more functional coatings than traditional approaches [87].

Protein–polymer conjugates are also becoming a part of our daily lives in the form of cosmetic and skin care products. A common player in this field is hyaluronic acid, used in skincare with its ability to hold water. Hyaluronic acid conjugates and cross-linked products are used for cosmetics and aesthetic processes [88,89]. Silk sericin, another common cosmetic ingredient, has many applications, from hydration and moisturization to UV protection. However, as it is not stable in water and cannot be solved in organic solvents, its usage in cosmetics is easier with sericin-PEG conjugation [90,91,92].

More suitable structural and functional protein–polymer conjugates are studied to create food ingredients. Especially, edible film coating has been tested to combine the individual traits of proteins and polysaccharides. In their 2014 study, Marquez et al. examined transglutaminase-crosslinked whey protein/pectin films. The study found that these films exhibited good water barrier properties and effectively reduced oil uptake in fried foods while maintaining the quality of baked goods [93]. In another study, Qu and Zhong created smaller fat globule mimetics (FGMs) with the help of casein-maltodextrin conjugates to encapsulate carbonate particles. They concluded that the FGMs with casein-maltodextrin conjugates are suitable for usage in food and have the advantage of reducing fat while increasing calcium content [94].

Incorporating protein moieties into polymeric oil absorbents is a promising approach to improve oil absorbency and increase the biodegradability of these materials [95,96]. The sensitivity of protein to microorganisms is an advantage for the biodegradability of these materials, but it also requires careful consideration during the design of protein-based conjugates. Site-specific polymer attachment to amine groups on protein can be a helpful strategy to achieve effective surface modification. Using waste resources, such as lignocellulosic biomass [97], hair, and feathers, for producing protein-based conjugates is an exciting development that could potentially reduce waste and provide sustainable alternatives to traditional oil-absorbing materials. However, it is essential to ensure that the extraction and processing of these materials are environmentally friendly and sustainable. Recent research on biopolymer conjugates is a promising area for improving environmental performance and reducing waste [98].

Protein–polymer conjugates hold significant potential in agricultural applications, offering innovative solutions for challenges such as efficient agrochemical delivery, soil conditioning, and crop protection. These conjugates can enable the controlled release of agrochemicals, reducing the frequency of application and minimizing environmental runoff. Amphiphilic protein–polymer conjugates, in particular, are highly effective for encapsulating hydrophobic pesticides, ensuring gradual and targeted release at the site of action, thereby reducing environmental contamination and improving pesticide efficiency. Additionally, these conjugates can be engineered to improve soil structure and water retention through soil conditioning agents, which enhance root development and nutrient availability. Beyond pest control and soil management, they may also be applied as carriers for bio-stimulants or fertilizers, increasing crop yield and reducing the environmental footprint of traditional farming practices. Establishing collaborations with agricultural scientists and industry stakeholders is crucial to fully explore these possibilities, optimize formulations for specific crops, and expand application networks, paving the way for sustainable and advanced agricultural technologies.

## 6. Conclusions and Perspectives

Hybrids hydrogels from protein–polymer conjugates create a modern approach to multifunctional and biocompatible material design for an enormous range of applications. Such hydrogels achieve structural stability with dynamic responsiveness by leveraging chemical crosslinking methods, such as click chemistry, amide bond formation, and Michael addition, along with physical interactions including hydrogen bonding and ionic interactions. Advanced methodologies, such as enzymatic crosslinking, self-assembly, and photopolymerization, make them more versatile by offering enhanced ways of controlling mechanical properties, swelling behavior, and biodegradability. The interplay of functional complexity at the level of proteins with tunable properties at the polymer level—for example, PEG—allows such hydrogels to resolve important challenges in drug delivery, tissue engineering, and biosensing. With their flexibility in encapsulation and bioactive delivery provoked by environmental stimuli, protein–polymer hydrogels have high prospects for the next generation biomaterials and hence demand further research and development.

Studies on protein–polymer conjugates, which reveal the structure-function relationship with the biomimetic principles required by interdisciplinary work, attract the attention of young academics who have just started their profession as well as expert researchers. These studies aim to create complex structures that support the relationship between polymer science and biology by increasing the efficiency of proteins via conjugation to polymers.

Despite the complexity of their structures, research on protein–polymer conjugates improved the stability and efficiency of this type of biomolecule. However, deriving form factors to represent the scattering of these structures is not always practical or realistic. Therefore, understanding and expressing the dynamics of newly formed structures is a crucial part of the studies in this field.

This review highlights the synthesis techniques, properties, and biomedical applications of protein–polymer conjugates. Their diverse functionalities and hybrid nature have paved the way for innovative biomedical uses, including drug delivery, tissue engineering, and biosensing. The ability to conjugate proteins with polymers enhances the stability, solubility, and biocompatibility of therapeutic proteins. In particular, PEGylation remains a dominant technique; however, concerns about its biodegradability and accumulation in vivo have prompted research into alternatives. Additionally, the exploration of various synthesis strategies, such as controlled radical polymerization (CRP) methods including ATRP and RAFT, has facilitated the creation of well-defined and functional protein–polymer conjugates.

Future studies should also explore stimuli-responsive hydrogels, which can respond to environmental triggers for on-demand therapeutic release. New developments in site-specific conjugation techniques are anticipated to reduce immunogenicity and increase targeting precision, further enhancing the biomedical utility of these systems. The ongoing integration of advanced materials, conjugation strategies, and responsive designs will continue to drive the evolution of next-generation biomaterials for healthcare applications.

In the future, conducting a more in-depth analysis of the structure-function relationship in protein–polymer conjugates and how to increase the usability of these structures in biological applications is of high importance. In addition, the development of new and advanced characterization techniques can provide significant improvements in the design and synthesis of protein–polymer conjugates.

An interdisciplinary approach to the study of protein–polymer conjugates may offer breakthrough solutions for biotechnology and drug development in the future. Therefore, the efforts of researchers and young academics working in this field will significantly contribute to the improvement of human health.

## Figures and Tables

**Table 1:** Polymers used in protein–polymer conjugates.

Polymer	Advantages	Disadvantages
PEG	High solubility, biocompatibility, and ability to extend protein half-life	Non-biodegradable, polydisperse
HPMA	Enhanced drug delivery, versatile applications	Complex synthesis methods
Dextran	Biocompatible, biodegradable	Lower mechanical strength
PGA	Biodegradable, enzymatically cleavable	Limited scalability

**Table 2:** Overview of synthesis methods for protein–polymer conjugates.

Synthesis Technique	Description	Advantages	Limitations	Biomedical Relevance
**Controlled Radical Polymerization** (**CRP**)	General method using radicals for precise polymer growth	Low polydispersity, tunable properties	Requires specific initiators and control agents	Smart hydrogels, drug delivery
ATRP	Uses halogenated initiators with metal catalysts	Precise chain control, low polydispersity	Requires expensive catalysts and controlled conditions	Drug carriers, responsive hydrogels
RAFT	Chain transfer agents regulate radical polymerization	High versatility, compatibility with diverse monomers	Chain transfer agent optimization needed	Biodegradable polymers, controlled-release systems
**Grafting-to**	Attaches pre-formed polymers to functional proteins	High yields, straightforward modification	Steric hindrance limits efficiency	PEGylated proteins, enzyme stabilization
**Grafting-from**	Polymerization initiates from protein-linked sites	Site-specific polymer growth, improved purification	Complex initiation control	Amphiphilic protein–polymer micelles
**Grafting-through**	Biomolecules participate as monomers in polymerization	Integrated conjugate networks	Requires biomolecule functionalization	DNA–protein conjugates, nanoparticle systems
**PEGylation**	Attaches PEG to proteins for stability and solubility enhancement	Reduced immunogenicity, extended half-life	Polydispersity, non-biodegradability	Therapeutic protein conjugates, drug delivery
Classical PEGylation	Nucleophilic reaction with amino or thiol groups	Simple and efficient	Limited site specificity	PEG-protein therapeutics
Bridging PEGylation	Uses disulfide bonds for linking	Maintains protein functionality	Sensitive to reducing conditions	Sustained-release drug carriers
Enzymatic PEGylation	Targets glutamine or glycoproteins	High site specificity, homogeneous products	Limited by enzyme availability	Uniform PEGylated proteins
**Site-Specific Conjugation**	Binds polymers to defined protein sites	Enhanced control of bioactivity	Requires functionalized polymers	Targeted drug delivery, responsive hydrogels
Cysteine-targeted	Uses thiol-disulfide exchange	Selective conjugation, robust bonds	Limited by cysteine availability	Stabilized protein–polymer conjugates
Lysine-targeted	Amide bond formation with amino groups	High reactivity, versatile applications	Potential random conjugation	Long-circulating bioconjugates

**Table 3:** Materials used in the AGET ATRP system [34].

Material	Function
Modified BSA	Protein macroinitiator
Cu2+/bipyridine	Catalyst
2-hydroxyethyl methacrylate (HEMA)	Monomer
N, N’-methylene diacrylamide	Cross-linker
Ascorbic acid	Reducing agent

**Table 4:** Factors that can influence the cytotoxicity of polymer–protein conjugates.

Factor	Description
Polymer	Some polymers are cytotoxic due to chemical toxicity or physical interference with cell function. Others are biocompatible and non-toxic.
Protein	Some proteins are inherently cytotoxic due to chemical toxicity or immune stimulation. Others are non-toxic.
Conjugation method	Conjugation methods that involve chemical crosslinking or toxic reagents can introduce cytotoxic groups into the material. Gentle methods that rely on chemical reactions or physical entrapment can reduce cytotoxicity.
Size and shape	Larger conjugates and those with irregular shapes may be more cytotoxic due to their ability to entrap or aggregate cells or disrupt cell function physically.
Delivery method	Some delivery methods may be more cytotoxic than others due to the stresses of injection or the presence of foreign substances in the delivery vehicle.

**Table 5:** Examples of Albumin-based polymer conjugates for drug delivery.

Type of Albumin	Polymer	Function
HAS	poly (β-aminoester Urethane)	Delivery of the Hyperuricemia-diseases treatment uricase (Uox)
HAS	(Dex-Mal) polymer	Delivery of the anti-cancer drug DOX
HAS	DEX (VS) + PEG	Delivery of the anti-cancer drug DOX
HAS	4-arm PEG-maleimide	Delivery of the anti-cancer protein TRAIL
BSA	Triblock copolymer (PCLA)	The encapsulation and delivery of pDNA vaccines
BSA	DMDOMA polymer	The delivery of CL075

## Data Availability

Not applicable.
